# Isolation and Analysis of the Nisin Biosynthesis Complex NisBTC: further Insights into Their Cooperative Action

**DOI:** 10.1128/mBio.02585-21

**Published:** 2021-10-05

**Authors:** Jingqi Chen, Oscar P. Kuipers

**Affiliations:** a Department of Molecular Genetics, Groningen Biomolecular Sciences and Biotechnology Institute, University of Groningen, Groningen, the Netherlands; University of Washington

**Keywords:** *Lactococcus lactis*, nisin biosynthesis machinery, alternating binding mechanism, full-length NisB, split NisB, NisA-NisB interaction

## Abstract

Nisin is synthesized by a putative membrane-associated lantibiotic synthetase complex composed of the dehydratase NisB, the cyclase NisC, and the ABC transporter NisT in Lactococcus lactis. Earlier work has demonstrated that NisB and NisT are linked via NisC to form such a complex. Here, we conducted for the first time the isolation of the intact NisBTC complex and NisT-associated subcomplexes from the cytoplasmic membrane by affinity purification. A specific interaction of NisT, not only with NisC but also with NisB, was detected. The cellular presence of NisB and/or NisC in complex with precursor nisin (NisA) was determined, which shows a highly dynamic and transient assembly of the NisABC complex via an alternating binding mechanism during nisin dehydration and cyclization. Mutational analyses, with cysteine-to-alanine mutations in NisA, suggest a tendency for NisA to lose affinity to NisC concomitant with an increasing number of completed lanthionine rings. Split NisBs were able to catalyze glutamylation and elimination reactions in an alternating way as efficiently as full-length NisB, with no significant influence on the following cyclization and transport. Notably, the harvest of the leader peptide in complex with the independent elimination domain of NisB points to a second leader peptide binding motif that is located in the C-terminal region of NisB, giving rise to a model where the leader peptide binds to different sites in NisB for glutamylation and elimination. Overall, these combined studies provide new insights into the cooperative biosynthesis mechanism of nisin and thereby lay a foundation for further structural and functional characterization of the NisBTC complex.

## INTRODUCTION

Ribosomally synthesized and posttranslationally modified peptides (RiPPs) form a large group of genetically encoded natural products with a vast range of biological properties, including antimicrobial activity (1). RiPP precursor peptides are typically composed of a C-terminal core peptide, where the posttranslational modifications (PTM) take place, and an N-terminal leader peptide, which serves as an allosteric effector to activate the biosynthetic enzymes, aids their secretion and often keeps the maturing peptide inactive (2, 3). The family of lanthipeptides, particularly lantibiotics with antimicrobial activities, is gaining interest as a potential source of antibiotics. Lanthipeptides have been divided into four classes (I to IV) based on distinct biosynthetic enzymes that carry out the dehydration and cyclization reactions (4, 5). Recently, lanthidins were proposed to be class V lanthipeptides that are made via a biosynthetically distinct pathway (1, 6).

**FIG 1:**
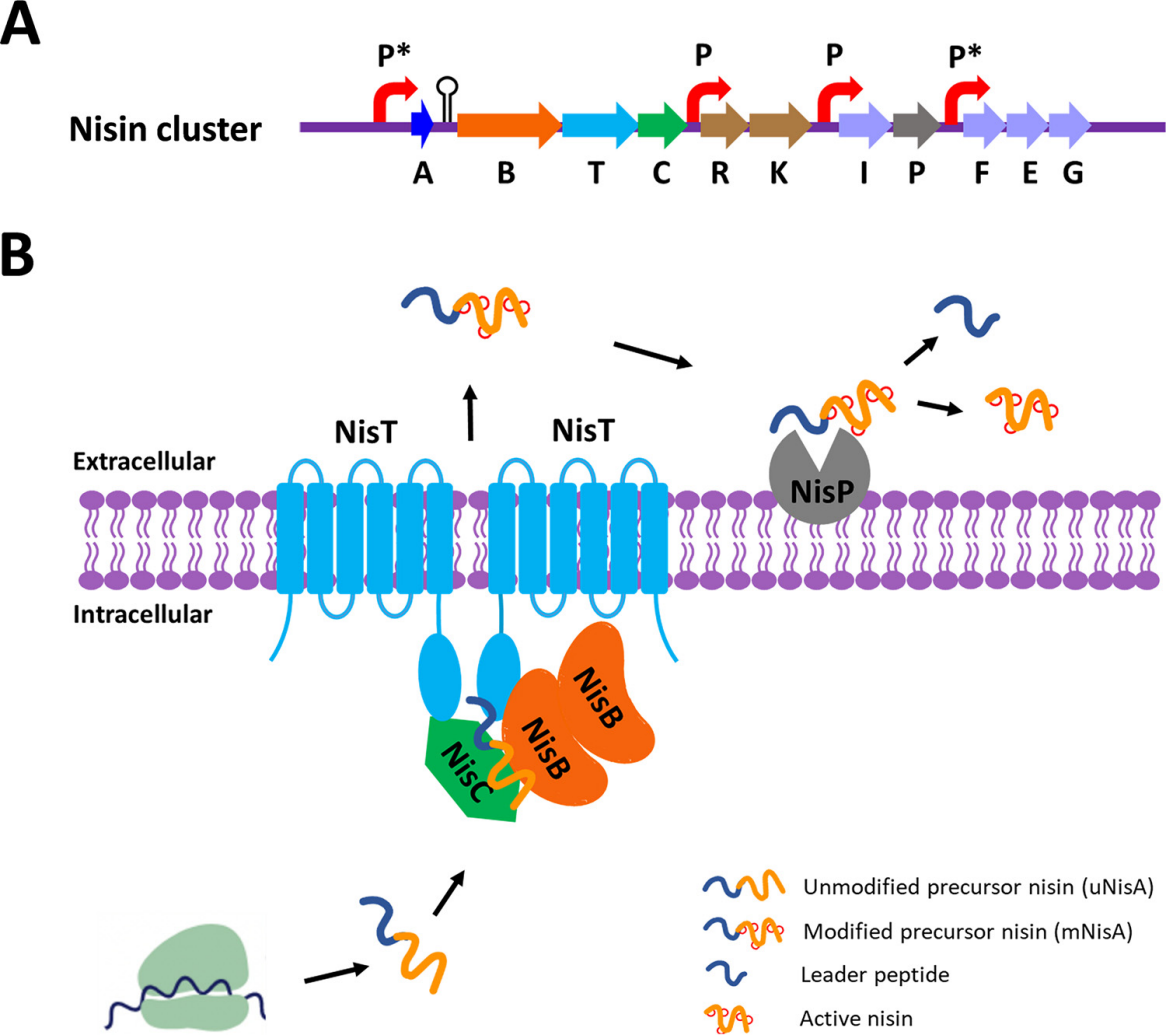
Graphical representation of the presumed biosynthesis of nisin in Lactococcus lactis. (A) The nisin gene cluster encodes precursor nisin (NisA) and proteins involved in modification (NisB and NisC), transport (NisT), regulation (NisR and NisK), and immunity (NisI and NisFEG). P*, the promoter is activated by extracellular nisin. P, constitutive promoter. (B) The process of nisin maturation and transport. The dehydratase NisB converts serine and threonine residues in the core peptide of unmodified NisA (uNisA) into dehydroalanine and dehydrobutyrine, respectively. The cyclase NisC catalyzes the addition of a thiol group in cysteine to an N-terminally located dehydroamino acid, resulting in the characteristic lanthionine rings. The ABC transporter NisT exports the fully modified NisA (mNisA) outside the cells, where the serine protease NisP extracellularly removes the leader peptide, releasing active nisin. A membrane-associated multimeric biosynthesis complex consisting of NisB, NisC, and NisT has been proposed for nisin maturation and transport.

Commonly, class I lanthipeptides are organized in a biosynthetic gene cluster encoding the precursor peptide (LanA), modification enzymes (LanB and LanC), an ABC transporter (LanT), a protease (LanP), a two-component regulation system (LanR and LanK), and an immunity system (LanI and LanFEG) (7). The mechanisms involved in modification, secretion, immunity, and regulation of LanA have been relatively well studied. Class I lanthipeptides are modified by two different PTM enzymes, a lantibiotic dehydratase, LanB (8–11), and a lantibiotic cyclase, LanC (12–14). The enzyme LanB dehydrates specifically serine or threonine residues via glutamylation and elimination reactions, whereas LanC catalyzes the thioether ring formation of the dehydrated amino acid and a C-terminally located cysteine residue within the core peptide via a Michael addition reaction (12). This results in the formation of lanthionine (from Ser) or (methyl)lanthionine (from Thr) rings, which are crucial for the activity as well as stability of the peptides. Subsequently, the fully modified precursor peptide is exported to the exterior by the ABC transporter LanT (15–17), followed by the cleavage of the leader peptide by the extracellularly located protease LanP, to release the active lantibiotics (18, 19). In some cases, i.e., for subtilin biosynthesis, a specific protease, LanP, is deficient (20). Some of the general proteases then take over this role, e.g., AprE, WprA, Vpr, and Bpr, in Bacillus subtilis (20, 21). Nisin, a class I lanthipeptide produced by Lactococcus lactis, is one of the best-studied lantibiotics. Its biosynthesis process, involving modification, transport, and leader peptide processing, has been schematically shown in Fig. 1 (see also Fig. S1 in the supplemental material).

10.1128/mBio.02585-21.1 FIG S1 Posttranslational modification of precursor nisin (NisA). (A) Serines and threonines in the core peptide of unmodified NisA are dehydrated by NisB. The resulting dehydrated NisA contains dehydroalanines (*Dha*) and dehydrobutyrines (*Dhb*) (highlighted in blue). Cysteines in the core peptide of NisA are highlighted in yellow. (B) Dehydrated residues, in turn, are specifically coupled to cysteines forming five thioether rings (A, B, C, D, and E), comprising one lantionine and four methyllantionines, generating fully modified NisA. (C) Fully modified NisA is transported by a dedicated ABC transporter NisT. (D) The leader peptide is cleaved off by extracellular protease NisP releasing active nisin. Download FIG S1, TIF file, 0.6 MB. Copyright © 2021 Chen and Kuipers. 2021 Chen and Kuipers. https://creativecommons.org/licenses/by/4.0/ This content is distributed under the terms of the Creative Commons Attribution 4.0 International license.

It has been proposed that the dehydratase, the cyclase, and the ABC transporter assemble as a cytoplasmic membrane-associated multimeric biosynthesis complex, LanBTC, for the maturation and transport of lantibiotics. Nisin modification enzymes, i.e., the dehydratase NisB and the cyclase NisC, have been demonstrated to be present at the cytoplasmic membrane in L. lactis (22). Using coimmunoprecipitation and a yeast two-hybrid screen, a molecular interaction between NisB and NisC, as well as NisC and NisT, which exports nisin from the cell, was detected, suggesting the existence of a nisin biosynthesis-associated NisBTC complex at the cell membrane of L. lactis producing nisin (23). Recently, the influence of the modification enzymes NisB and NisC on the transport kinetics of NisT was evaluated *in vivo*, and an interaction of NisT with NisB besides the interaction between NisT and NisC was detected *in vitro* (24). The subcellular localization and the assembly process of the intact nisin biosynthesis complex were described, proposing a model for polar modification and transport of nisin in L. lactis (4). For subtilin biosynthesis, SpaB was shown to localize at the cytoplasmic membrane of B. subtilis (25) and to interact with SpaC when both proteins were overexpressed in Escherichia coli (9). Moreover, SpaB, SpaC, and SpaT have been reported to assemble as a membrane-associated SpaBTC complex in B. subtilis (26). For class II lanthipeptides, the enzyme NukM and the ABC transporter NukT were proven to assemble as a membrane-located multimeric protein complex, NukMT, for the production of Nukacin ISK-1 in Staphylococcus warneri ISK-1 by yeast two-hybrid assays and surface plasmon resonance (SPR). NukM expressed heterologously in Staphylococcus carnosus TM300 was located at the cytoplasm membrane even when NukT was not present (27). Besides the putative LanBTC complex, a few subcomplexes mainly involved in the modification of LanA have been well characterized. Extensive studies on the NisAB complex have been reported, i.e., structure analysis (28, 29), NisA-NisB interaction (30–32), and substrate specificity for NisB (8, 33). The interactions between NisA in different modification states and NisC have been evaluated *in vitro* (34). A pulldown assay demonstrated that NisB and NisC were copurified with an engineered His-tagged NisA (32). The assembly of the NisABC complex was conducted *in vitro*, and the complex was suggested to comprise a NisB dimer, a monomer of NisC, and one NisA monomer (35). In spite of these studies, successful isolation of the complete LanBTC complex has not been reported yet, which limits the investigation into the mechanism of lanthipeptide modification and transport as a combined process.

In this study, we employed the strategy of pulldown to isolate the intact nisin biosynthetic machinery, NisBTC, as well as its various subcomplexes from the cytoplasmic membrane of nisin-producing strains. Regardless of the peptide transport, the corresponding intensity of the cellular nisin modification-related complexes was determined. Applying the mutagenesis in the substrate NisA, the influence of ring formation in the core peptide on the interaction between the substrate and the enzymes was evaluated. Notably, we demonstrated that NisB could be split into two independent domains, which did not lead to any effect on the dehydration, cyclization, and secretion of NisA. Our work provides direct evidence for the existence of the nisin biosynthesis machinery in nature and elucidates the interactions among its components, including subdomains of NisB.

## RESULTS

### Direct evidence for the presence of NisBTC at the cytoplasmic membrane.

**FIG 2:**
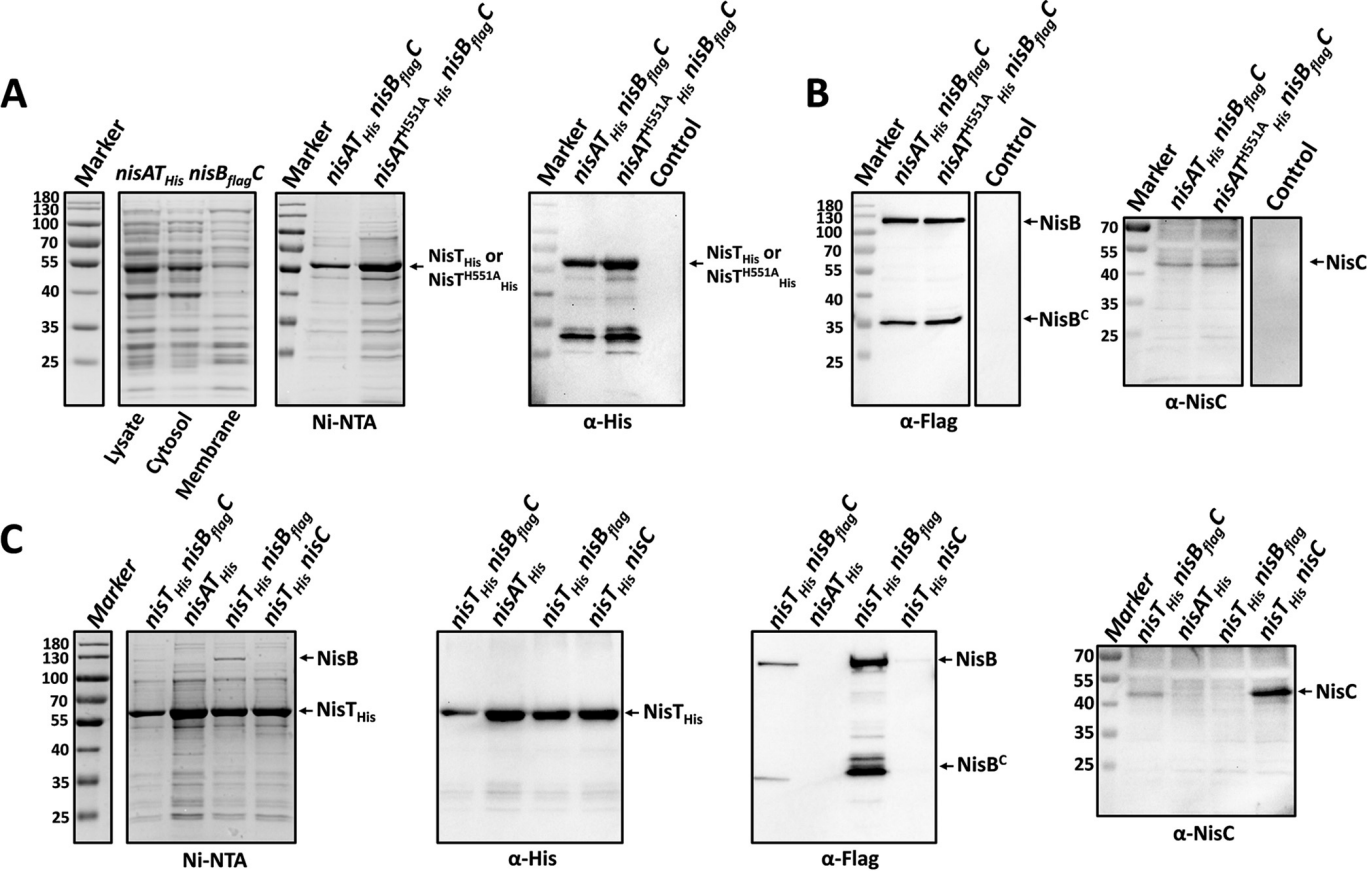
Isolation of the NisBTC complex and its NisT-associated subcomplexes. (A) Purification of NisT from the cytoplasmic membrane. NisA, NisB_flag_, NisC, and NisT_His_/NisT^H551A^_His_ were coexpressed in L. lactis NZ9000. The elutions were analyzed by SDS-PAGE (left lane) and Western blotting with anti-His antibody (right lane). The band at ∼30 kDa in the Western blot was a result caused by unspecific binding. Control, NZ9000/pNZE3-*nisAT* pIL3-*nisB_flag_C*. (B) Copurification of NisB and NisC with NisT. The elutions of NisT purification were subjected to Western blotting with anti-Flag and anti-NisC antibodies. Control, NZ9000/pNZE3-*nisAT* pIL3-*nisB_flag_C*. (C) Different expression combinations of NisA, NisB_flag_, NisC, and NisT_His_ in L. lactis NZ9000. For membrane protein purification, the cell membrane was collected by ultracentrifugation and solubilized by *n*-dodecyl-β-d-maltoside (DDM). The proteins were purified from the solubilized membrane by affinity purification using Ni-NTA agarose. The elutions were analyzed by glycine SDS-PAGE followed by Coomassie G-250 staining or Western blotting using anti-His, anti-Flag, and anti-NisC antibodies as indicated. NisT_His_, NisT C-terminally labeled by a 6×His tag. NisT^H551A^_His_, the mutation H551A was introduced in NisT_His_. NisB_flag_, NisB C-terminally labeled by a Flag tag. NisB^C^, the C-terminal product of degraded NisB. NisT_His_/NisT^H551A^_His_ size, 70 kDa. NisB_flag_ size, 120.2 kDa. NisC size, 47.9 kDa.

Although the nisin modification and transport machinery NisBTC has been proposed for a long time, the direct isolation of such a complex has not been conducted yet (7, 23). This is due in part to the presumed transient existence of this complex and its low expression level and low complex stability in the native nisin producer. Here, we present the isolation of the intact nisin biosynthesis machinery NisBTC through copurification with the dedicated ABC transporter NisT from the cell membrane. For this purpose, we overexpressed nisin biosynthesis-associated proteins in L. lactis NZ9000 harboring two multicopy plasmids: pNZE3-*nisAT_His_* carrying the genes encoding precursor nisin (NisA) and C-terminally 6×His-tagged NisT and pIL3-*nisB_flag_C* expressing the modification enzymes NisB, labeled by Flag tag at its C terminus, and tag-free NisC. All four genes were under the control of the nisin-inducible promoter P*_nisA_* (36). The tags did not prevent the modification and secretion of NisA, which was confirmed by Western blotting and antimicrobial activity assay (Fig. S2A and B). The cell membrane was separated, solubilized in the detergent *n*-dodecyl-β-d-maltoside (DDM), and subjected to Ni-NTA purification. SDS-PAGE and Western blotting with anti-His antibody (Fig. 2A) showed that NisT_His_ was purified with a migrated band smaller than the calculated size (70 kDa), displaying a higher mobility, similar to other membrane proteins. Compared to tag-free NisT, the expected shift of protein size (∼26 kDa) was observed from the isolated fusion protein NisT^sfGFP^_His_, where NisT was C-terminally labeled by the superfolder green fluorescent protein (sfGFP) coupled with a 6×His tag (Fig. S3A to C), confirming the correct isolation of NisT described above. The same elutions of NisT_His_ purification were applied in Western blot analysis with anti-Flag and anti-NisC antibodies (Fig. 2B). Both NisB_Flag_ (120.2 kDa) and NisC (47.9 kDa) were copurified in this way. The substrate NisA was not detected in the elution by Western blotting, probably owing to its weak affinity to NisT and the low yield of its copurification (data not shown). When the histidine residue at position 551 located in the H-loop of the nucleotide binding domain (NBD) of NisT was changed to alanine, the ATPase activity of the H551A mutant was severely reduced, and the secretion of nisin was nearly abolished (24). In our study, the purification of NisT^H551A^_His_ from the cell membrane still pulled down the enzymes NisB_Flag_ and NisC, and the copurification yields of the enzymes were not affected by the mutation (Fig. 2B). This indicates that the association of NisT with NisB and NisC is unrelated to its ATPase activity. Altogether, the NisBTC complex was successfully isolated from the cytoplasmic membrane of L. lactis, producing fully modified precursor nisin, which exhibited antimicrobial activity after removing the leader peptide (Fig. S2A and B). Here, direct evidence for the presence of the membrane-associated multimeric nisin synthetase NisBTC complex in L. lactis was presented.

10.1128/mBio.02585-21.2 FIG S2 Determination of the functionality of the NisABTC complex with tags (sfGFP/6xHis/Flag-tag). (A) Western blot analysis of secreted NisA in the supernatant of cell culture. Antibody, anti-leader. (B) Antimicrobial activity assay for the supernatant of cell culture. The supernatant of cell culture was incubated at 30°C with purified NisP to remove the leader peptide. Indicator strain, Micrococcus flavus. Download FIG S2, TIF file, 1.2 MB. Copyright © 2021 Chen and Kuipers. 2021 Chen and Kuipers. https://creativecommons.org/licenses/by/4.0/ This content is distributed under the terms of the Creative Commons Attribution 4.0 International license.

10.1128/mBio.02585-21.3 FIG S3 Purification of the fusion protein NisT^sfGFP^_His_. (A) The fusion protein NisT^sfGFP^ was C-terminally tagged by a 6× His tag, termed NisT^sfGFP^_His_. (B) SDS-PAGE of the purified NisT^sfGFP^_His_. NisT^sfGFP^_His_ was coexpressed with NisA, NisB_flag_, and NisC in L. lactis NZ9000. The cell membrane was separated by ultracentrifugation and solubilized by *n*-dodecyl-β-d-maltoside (DDM). The proteins were purified from the solubilized membrane by affinity purification using Ni-NTA agarose. Elution 1, elution 2, and elution 3 were three differentially eluted fractions from Ni-NTA purification. The elutions were analyzed by 10% glycine SDS-PAGE followed by Coomassie G-250 staining. (C) Western blot analysis for elution 2. Antibody, anti-sfGFP antibody. The size of NisT^sfGFP^_His_ is ∼96 kDa. Download FIG S3, TIF file, 1.0 MB. Copyright © 2021 Chen and Kuipers. 2021 Chen and Kuipers. https://creativecommons.org/licenses/by/4.0/ This content is distributed under the terms of the Creative Commons Attribution 4.0 International license.

To get insight into the interactions between the components of the intact NisBTC machinery, the isolation of NisT-associated subcomplexes was performed in the absence of corresponding genes (Fig. 2C). When NisA was deficient, NisB_Flag_ and NisC were still coeluted with NisT_His_, implying that the formation of NisBTC does not depend on the presence of the substrate peptide. When NisT_His_ was coexpressed with NisB_Flag_ or NisC, Western blot analysis showed that the NisTB and NisTC complexes were harvested, which indicates that NisT directly interacts with not only NisC but also NisB. This is in line with a recent *in vitro* interaction study (24). From SDS-PAGE, a clear band of copurified NisB could be seen, but NisC was not. The molecular affinity of NisT to NisB seems to be higher than that to NisC. This is supported by the role of NisB as a recruiter to target NisT to the old cell poles during the *in vivo* dynamic assembly of NisBTC (4). When NisT_His_ was coproduced with only the substrate peptide, NisA could not be detected in the NisT_His_ elution by Western blotting (data not shown). Although it has been shown that NisA was unmodified and could be exported by NisT in the absence of NisBC, its secretion efficiency was extremely low (37). Perhaps the presumed weak affinity of unmodified NisA to NisT led to unsuccessful isolation of NisAT. Overall, both NisB and NisC enzymes directly interact with NisT to assemble the NisBTC complex, which does not need the stimulation of nisin transport through the cell membrane and the full ATPase activity of NisT.

Interestingly, except full-length NisB, an ∼30-kDa protein (NisB^C^) was found to be pulled down with NisT_His_ when determined using anti-Flag antibody (Fig. 2B and C). It has been reported that a truncated product, the N-terminal ∼90-kDa part of NisB, is present in the cytoplasm and at the cell membrane of L. lactis (22, 32). Hence, we assume that the observed ∼30-kDa band is the C-terminal part of NisB. Since the Flag tag was fused to the C terminus of NisB, the degraded N-terminal part of NisB was not detected but should be present in our case.

### Assembly of the NisABC complex, probably via an alternating binding mechanism for dehydration and cyclization.

Nisin contains several posttranslational modifications introduced by the maturation machinery NisBC in a proposed alternating manner (34, 38). Khusainov et al. performed pulldown assays resulting in the isolation of a predominant NisAB complex and very small amounts of NisAC complex, whereas the NisABC complex was not observed in a direct way (32). Reiners et al. demonstrated the assembly of NisABC *in vitro*, which was suggested to be formed only in the presence of the substrate (35). To characterize the assembly manner of the NisABC complex further, we separated the NisAB, NisAC, and NisABC complexes simultaneously from the cellular complex pool and determined their cellular intensity using size-exclusion chromatography (SEC).

**FIG 3:**
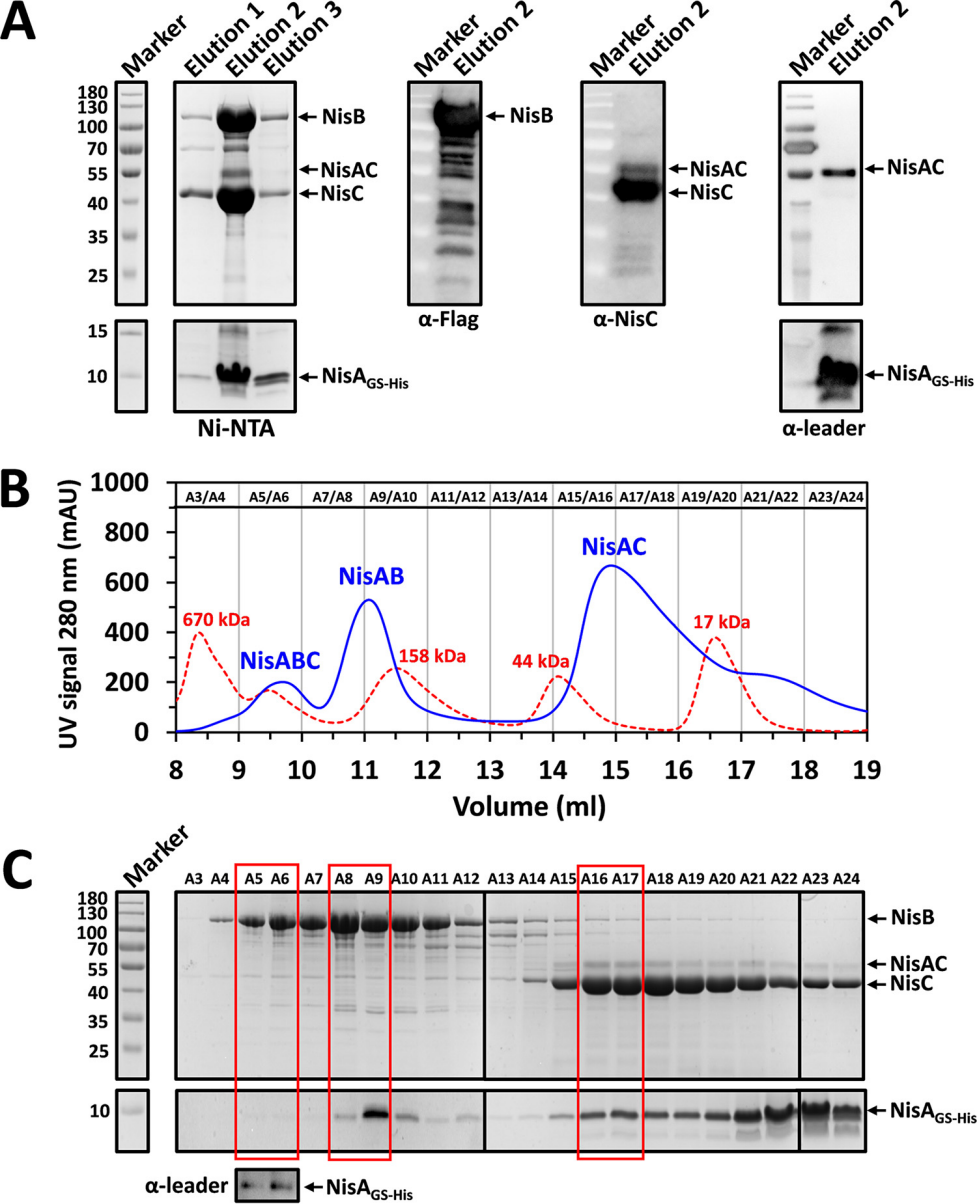
Analysis of the cellular intensity of the NisAB, NisAC, and NisABC complexes. NisA_GS-His_, NisB_flag_, and NisC were coexpressed in L. lactis NZ9000. The proteins were purified from the cell lysate by affinity purification using Ni-NTA agarose. During Ni-NTA purification, 20 mM imidazole was used in the wash buffer. (A) Isolation of the NisAB, NisAC, and NisABC complexes. Elution 1, elution 2, and elution 3 were three different eluted fractions from the Ni-NTA purification. All three elutions were analyzed by 8% glycine and 16% tricine SDS-PAGE followed by Coomassie G-250 staining, while elution 2 was applied in Western blotting using anti-leader, anti-Flag, and anti-NisC antibodies as indicated. (B) Size-exclusion chromatography (SEC) analysis for elution 2 of Ni-NTA purification. The SEC column ENrich SEC 650 10/300 was calibrated using gel filtration standard (red dotted peaks), which is composed of thyroglobulin (670 kDa), ϒ-globulin (158 kDa), ovalbumin (44 kDa), myoglobin (17 kDa), and vitamin B_12_ (13.5 kDa), at 4°C while measuring absorbance at 280 nm (mAU) per ml elution volume. Eluted peaks (blue peaks) of the sample are indicated with the corresponding complexes (NisABC, NisAB, and NisAC), according to size estimation (Fig. S5). (C) Elution fractions from SEC were analyzed by 8% glycine and 16% tricine SDS-PAGE or Western blotting using anti-leader antibody. Red boxes represent the fractions containing corresponding complexes. NisA_GS-His_, NisA C-terminally extended with a factor Xa sequence, a flexible liner, and a 6×His tag. NisB_flag_, NisB C-terminally labeled by a Flag tag. NisAC, NisC in complex with NisA_GS-His_. NisA_GS-His_ size, 7.7 kDa. NisB_flag_ size, 120.2 kDa. NisC size, 47.9 kDa.

The substrate peptide NisA was C-terminally extended with the sequence IEGRGSGGGGSHHHHHH, termed NisA_GS-His_ in short. GSGGGGS was used as a flexible linker to keep a space between the core peptide and the 6×His tag to facilitate the purification. IEGR is the factor Xa cleavage sequence that was introduced in front of the linker and the 6×His tag with the purpose of removing the tag in a later step, when desired. The engineered substrate and both enzymes were overexpressed using a two-plasmid expression system in the strain in the deficiency of the ABC transporter NisT so that the peptide NisA_GS-His_ could be continuously enriched in cells, with an expected improvement of the yield of protein complexes. NisA_GS-His_ was harvested in abundance by nickel-nitrilotriacetic acid (Ni-NTA) purification. SDS-PAGE combined with Western blotting using anti-Flag and anti-NisC antibodies showed that large amounts of NisB and NisC were copurified with a similar yield in this way (Fig. 3A). Three clearly separated elution peaks were observed from the subsequent SEC analysis (Fig. 3B). To identify the proteins in the elutions corresponding to the peaks, the elution fractions from the SEC experiment were subjected to SDS-PAGE analysis in conjunction with Western blotting (Fig. 3C). Obviously, the second and third peaks were found to be the NisAB and NisAC complexes, respectively. For the first peak, the estimation of the protein complex was conducted by creating the calibration curve using a gel filtration standard (Bio-Rad) (Fig. S5A). The estimated size was close to the theoretical size of the NisABC complex, supporting that the first peak was NisABC (Fig. S5B). Within the cells, NisAB and NisAC were present at a relatively high level, but the intensity of the modification complex NisABC was much lower. Coupled with the proposed alternating manner of nisin modification by NisB and NisC, this observation suggests an alternating binding mechanism for the assembly of the NisABC complex *in vivo*.

10.1128/mBio.02585-21.5 FIG S5 Calibration curve used to estimate molecular weight for the protein complex. (A) Calibration curve. Size exclusion chromatography column ENrich SEC 650 10/300 was calibrated using gel filtration standard (Bio-Rad), which is composed of thyroglobulin (670 kDa), ϒ-globulin (158 kDa), ovalbumin (44 kDa), myoglobin (17 kDa), and vitamin B12 (13.5 kDa). The calibration curve was plotted using the gel-phase distribution coefficient (*K*_av_) versus logarithm of the molecular weight (log MW). *K*_av_ = (*V*e − *V*o)/(*V*c − *V*o), where *V*e = elution volume, *V*o = column void volume, *V*c = geometric column volume. Straight line is the calibration curve calculated from the data for molecular weight standards (*R^2^* = 0.9486). Blue circles correspond to the positions of *K*_av_ values for the gel filtration standard. (B) Estimation of molecular weight of the protein complexes. Red triangles correspond to the positions of *K*_av_ values for the purified protein complexes (Fig. 3B). The equation, *y* = −0.2993*x* + 1.832 from the calibration curve, was used to calculate the experimental molecular weights. The estimated molecular weight of the presumed NisABC complex, 314.21 kDa. The theatrical molecular weight of the NisABC complex (two NisA_GS-His_, two NisB_flag_, and one NisC), 303.7 kDa. Download FIG S5, TIF file, 0.6 MB. Copyright © 2021 Chen and Kuipers. 2021 Chen and Kuipers. https://creativecommons.org/licenses/by/4.0/ This content is distributed under the terms of the Creative Commons Attribution 4.0 International license.

### NisA tends to lose affinity to NisC with an increasing number of finished lanthionine rings in the core peptide.

The core peptide of the wild-type NisA contains five cysteine residues, giving rise to five (methyl)lanthionine rings (A, B, C, D, and E) due to the modification by NisB and NisC (Fig. S1A and B). To evaluate the influence of ring formation on the interplay of the enzymes with the substrate, five peptide variants differing in the number of (methyl)lanthionine rings within the core peptide of NisA_GS-His_ were created by mutating cysteine residues to alanine (Table 1). Here, the peptide CCCCC represents NisA_GS-His_ with five rings, A to E, while the variant AAAAA does not contain any ring. The peptides CCCCA, CCCAA, CCAAA, and CAAAA harbor rings A-D, A-C, AB, and A, respectively. The peptide variants as well as the NisB and NisC enzymes were overexpressed in the cells, ensuring that the variant peptides were dehydrated and the lanthionine rings were formed when cysteine residue was still available.

**TABLE 1:**
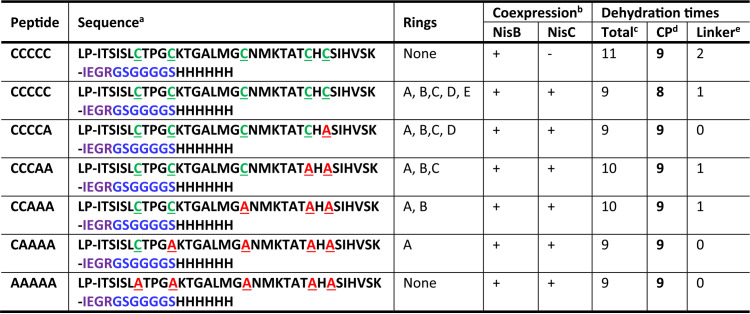
Dehydration of the engineered peptide variants differing in the number of (methyl)lanthionine rings

^*a*^Cysteine residue (green) in the core peptide was changed to alanine (red) to generate the peptide variants with different rings. LP, the leader peptide of NisA. IEGR, factor Xa sequence. GSGGGGS, flexible linker sequence. HHHHHH, 6xHis-tag.

^*b*^+, protein was expressed; −, protein was not expressed.

^*c*^The MALDI-TOF MS analysis for the peptides is shown in Fig. S4.

^*d*^CP, the core peptide of NisA. The dehydration of CP was determined after incubation with factor Xa protease.

^*e*^The dehydration times in the linker were calculated by the total dehydration times minus the dehydration times of CP.

10.1128/mBio.02585-21.4 FIG S4 MALDI-TOF MS analysis of NisA_GS-His_ variants differing in the number of (methyl)lanthionine rings. CCCCC, CCCCA, CCCAA, CCAAA, CAAAA, and AAAAA are NisA_GS-His_ peptides that harbor five, four, three, two, one, and no cysteine residues in the core peptide, respectively. The peptides were coexpressed with NisB/NisBC. The serine-containing linker and the 6×His tag were removed by incubation with factor Xa protease. Download FIG S4, TIF file, 1.8 MB. Copyright © 2021 Chen and Kuipers. 2021 Chen and Kuipers. https://creativecommons.org/licenses/by/4.0/ This content is distributed under the terms of the Creative Commons Attribution 4.0 International license.

Matrix-assisted laser desorption–time-of-flight mass spectrometry (MALDI-TOF MS) showed that the peptide CCCCC purified from the cells with coexpression of NisB and NisC was modified by NisB with nine dehydrations (Table 1, Fig. S4). Since the employed linker contained two extra serine residues, which are usually dehydrated in the core peptide of the wild-type NisA, the distribution of the observed nine dehydrations in the engineered peptide was unclear. Hence, the C-terminal linker and 6×His tag were removed by incubation with the factor Xa protease. The resulting NisA-IEGR was found to be dehydrated eight times in the core peptide, which implied that the ninth dehydration occurred in the linker. It has been revealed that normally NisA is dehydrated eight times and Ser29 is not dehydrated in the wild-type situation (32, 38). Thus, the attachment of extra sequence to the C terminus of NisA did not affect its dehydration in the core peptide, whereas when NisC was not present, we found the peptide CCCCC was dehydrated 11 times by NisB, with nine dehydrations in the core peptide and two in the linker. Nine dehydration reactions catalyzed by NisB in the core peptide were also observed in all the peptide variants, CCCCA, CCCAA, CCAAA, CAAAA, and AAAA, when both NisB and NisC were present. Only the dehydration extent of serine residues in the linker was not fully consistent (Table 1, Fig. S4). In the above-described cases, there is the similarity that the lanthionine ring E is deficient in these peptides, which likely allows Ser29 to be dehydrated. These results support the hypothesis that two intertwined rings of nisin protect Ser29 against dehydration (38) and also reinforce the relaxed substrate specificity of NisB. Notably, the escape of dehydration on Ser29 reflects the coordination of dehydration reaction and ring formation. Once Thr25 is dehydrated, NisC binds to NisA and catalyzes the cyclization between dehydrated Thr25 and free Cys28 prior to the dehydration reaction on Ser29 performed by NisB. This cooperative manner may also apply to other dehydration and cyclization reactions in the core peptide during the modification process, which also supports the above-proposed NisABC assembly via an alternating binding mechanism.

**FIG 4:**
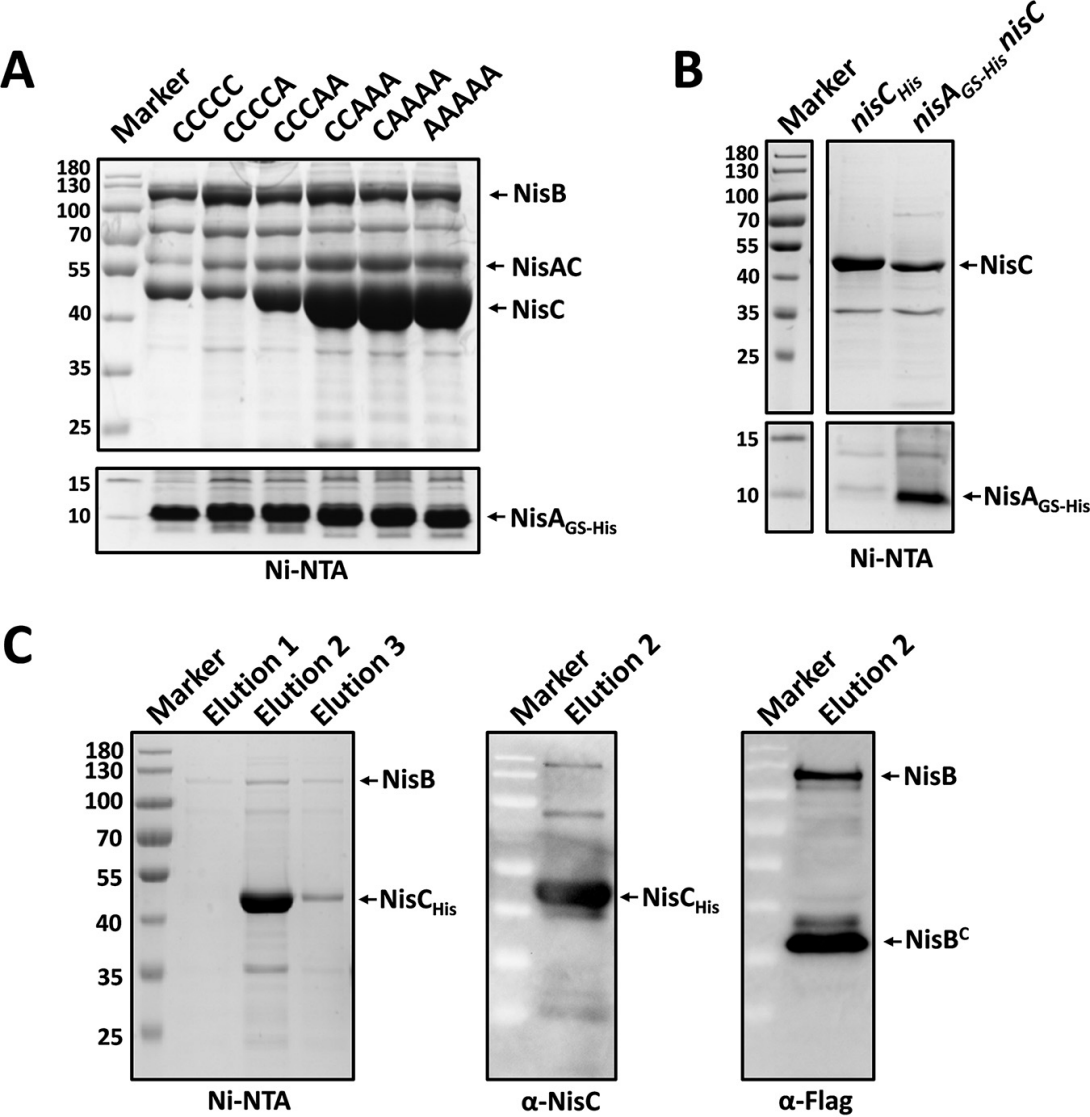
Interactions between the components of the nisin modification machinery. (A) Purification of NisA_GS-His_ variants with different rings formed in the core peptide. CCCCC, NisA_GS-His_ harbors five cysteine residues in the core peptide. CCCCA, NisA_GS-His_ contains four cysteine residues in the core peptide. CCCAA, NisA_GS-His_ harbors three cysteine residues in the core peptide. CCAAA, NisA_GS-His_ contains two cysteine residues in the core peptide. CAAAA, NisA_GS-His_ harbors one cysteine residue in the core peptide. AAAAA, NisA_GS-His_ does not contain a cysteine residue in the core peptide. The peptide sequences and the mutant residues are shown in Table 1. All peptides were coexpressed with the enzymes NisB_flag_ and NisC in L. lactis NZ9000. (B) Purification of unmodified NisA_GS-His_ in complex with NisC. NisA_GS-His_ was coexpressed with NisC in L. lactis NZ9000. (C) Isolation of the NisBC complex in the absence of NisA. NisC_His_ was coexpressed with NisB_flag_ in L. lactis NZ9000. Elution 1, elution 2, and elution 3 were three differentially eluted fractions from the Ni-NTA purification. In panels A to C, the proteins were purified from the cell lysate by affinity purification using Ni-NTA agarose. The elutions were analyzed by 8% glycine or 16% tricine SDS-PAGE followed by Coomassie G-250 staining and Western blotting using anti-Flag and anti-NisC antibodies. NisA_GS-His_, NisA C-terminally labeled by a factor Xa sequence, a flexible linker, and a 6×His tag. NisB_flag_, NisB C-terminally labeled by a Flag tag. NisB^C^, the C-terminal product of degraded NisB. NisA_GS-His_ size, 7.7 kDa. NisB_flag_ size, 120.2 kDa. NisC_His_ size, 48.7 kDa.

Pulldown assays demonstrated that both NisB and NisC were coeluted with all five peptide variants besides CCCCC (Fig. 4A), revealing that the presence of cysteine residues in the core peptide and the ring formation are not the prerequisites of the binding of the substrate peptide to the enzymes, especially NisC, and also probably not necessary for the assembly of the NisABC complex, as reported in a previous *in vitro* study (35). An increase of cysteine mutation in the core peptide did not result in a significant change of NisB copurification, while it largely enhanced the yield of NisC. This suggests that NisA with fewer rings possesses stronger affinity for NisC than NisA with more rings, likely facilitating the targeting of NisA being dehydrated to NisC for the formation of desired lanthionine rings in the wild-type situation. When NisB was absent, NisC in complex with unmodified NisA_GS-His_ was isolated (Fig. 4B, Fig. S6A), showing a quite strong affinity of NisA that does not contain any rings and is not even dehydrated to NisC. Based on these findings, we propose that NisA tends to lose affinity to NisC with increasing numbers of finished lanthionine rings in the core peptide, which eventually promotes the release of fully modified NisA from the modification machinery for the following export.

10.1128/mBio.02585-21.6 FIG S6 Purification of NisA in complex with modification enzymes. (A) Purification of unmodified NisA in complex with NisC. NisA_GS-His_ was coexpressed with NisC in L. lactis NZ9000, where NisB and NisT were deficient. Upper lane, SEC analysis of the elution after affinity purification. The single eluted peak is indicated with the NisAC complex. Lower lane, elution fractions from SEC were analyzed by 8% glycine and 16% tricine SDS-PAGE. Red box represents the fractions containing the NisAC complex. E2, elution 2 of the affinity purification. (B) Isolation of the NisAC complex by removing NisB using increased concentration of imidazole in the wash buffer. NisA_GS-His_, NisB_flag_, and NisC were coexpressed in L. lactis NZ9000. During Ni-NTA purification, 35 mM imidazole was used in the wash buffer. Upper lane, SEC analysis of the elution of Ni-NTA purification. Eluted peaks are indicated with the corresponding complexes (NisAB and NisAC). Lower lane, the elutions of Ni-NTA and SEC purification were analyzed by 8% glycine and 16% tricine SDS-PAGE followed by Coomassie G-250 staining. For SEC analysis, the sample was subjected to SEC on an ENrich SEC 650 10/300 column at 4°C while measuring absorbance at 280 nm (mAU) per ml elution volume. NisA_GS-His_, NisA C-terminally extended with a factor Xa sequence, a flexible liner, and a 6×His tag. NisB_flag_, NisB C-terminally labelled by a Flag tag. NisAC, NisC in complex with NisA_GS-His_. NisA_GS-His_ size, 7.7 kDa. NisB_flag_ size, 120.2 kDa. NisC size, 47.9 kDa. Download FIG S6, TIF file, 1.1 MB. Copyright © 2021 Chen and Kuipers. 2021 Chen and Kuipers. https://creativecommons.org/licenses/by/4.0/ This content is distributed under the terms of the Creative Commons Attribution 4.0 International license.

From SDS-PAGE and Western blot analysis (Fig. 3A and C and 4A), we noticed that some amounts of NisC were still present in complex with NisA after heat treatment, but NisB was not. Moreover, during Ni-NTA purification, an increase of the imidazole concentration (from 20 mM to 35 mM) in the wash buffer removed almost all NisB from the Ni column, but the majority of NisC remained attached to the Ni column via NisA. The NisAC complex was found to be the main content in the elution (Fig. S6B). These observations imply that NisAC is a quite stable protein complex and NisC possesses a stronger affinity to NisA than NisB, which may explain how NisA is transferred from NisB to NisC during the catalysis. A study reported that only the presence of unmodified and dehydrated precursor nisin triggers the formation of the NisBC complex (35). On the contrary, in our study, NisB as well as its degraded C-terminal part was definitely copurified with 6×His-tagged NisC in the absence of precursor nisin (Fig. 4C). In agreement with this, NisB and NisC have been reported to colocalize at the old cell poles when NisA is not present in L. lactis (4). Perhaps the *in vitro* environment lacks some unknown essential factors for the direct interaction of NisB with NisC.

### Split NisBs (glutamylation domain and elimination domain) are fully functional.

**FIG 5:**
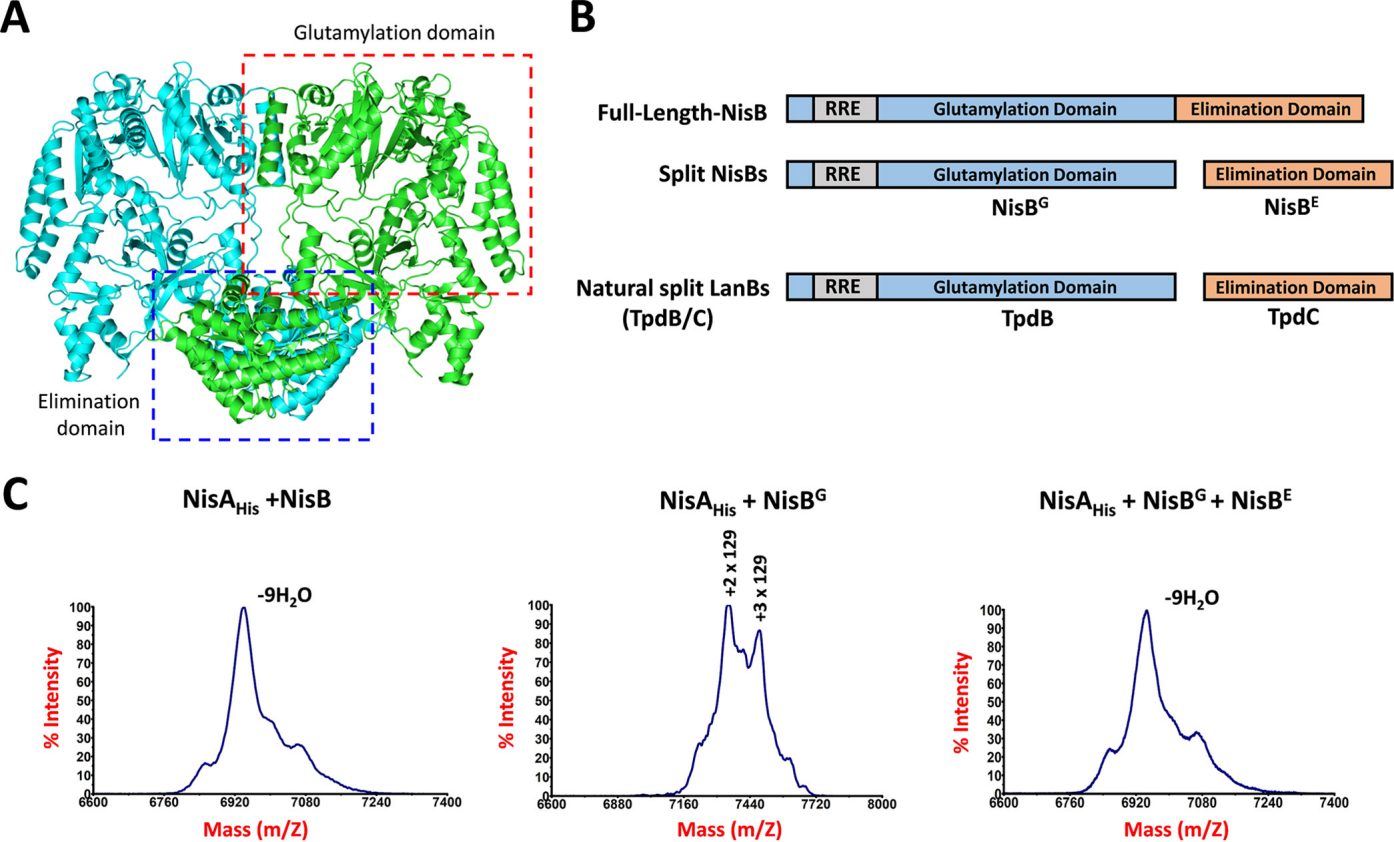
Functionality of split NisBs. (A) The crystal structure of the NisB homodimer (PDB accession no. 4WD9). One monomer is shown in cyan; the other monomer is shown in green. Each monomer (i.e., the green one) is composed of one glutamylation domain (red box) and one elimination domain (blue box). (B) Domain organization of full-length NisB, split NisBs, and split LanBs. NisB^G^, the glutamylation domain of NisB. NisB^E^, the elimination domain of NisB. TpdB, the glutamylation domain of split LanBs. TpdC, the elimination domain of split LanBs. RRE, RiPP precursor peptide recognition element. (C) MALDI-TOF MS analysis of NisA_His_. NisA_His_, NisA C-terminally labeled by a factor Xa sequence and a 6×His tag. NisA_His_ was coexpressed with full-length NisB, NisB^G^, or split NisBs (NisB^G^ and NisB^E^) in L. lactis NZ9000. The peptides were purified from the cell lysate by affinity purification using Ni-NTA agarose. The peptides with multiple 129 adducts are intermediates during dehydration reaction.

From the above-described data, we noticed that the putative ∼30-kDa C-terminal part of NisB was pulled down with both NisT and NisC. In conjunction with the previous finding that the ∼90-kDa N-terminal part of NisB is present in the cytoplasmic membrane of native producer cells (22), we hypothesize that a part of cellular NisB is divided into two truncated products under natural conditions. The crystal structure of the NisAB complex shows that NisB comprises an N-terminal 700-residue glutamylation domain and a C-terminal 300-residue elimination domain (Fig. 5A) (28). The sizes of both domains are highly identical to that of the corresponding truncated NisBs, as observed.

**FIG 6:**
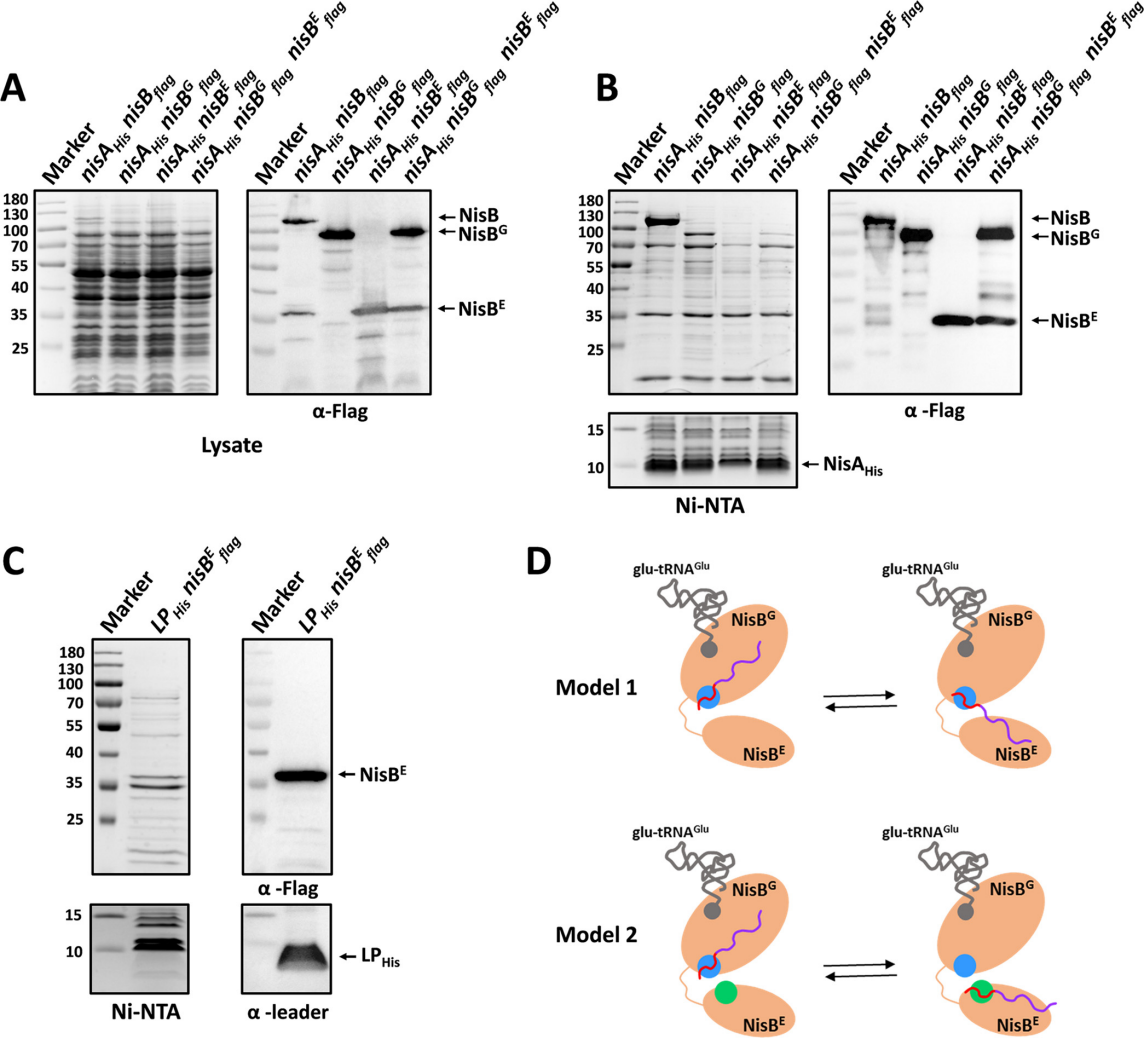
Interactions of NisA with split NisBs. (A) Detection of NisA and NisB/split NisBs in the cells by SDS-PAGE (left lane) and Western blot (right lane). NisA_His_, NisA C-terminally labeled by a factor Xa sequence and a 6×His tag. (B) Analysis of the elutions from pulldown assays by SDS-PAGE (left lane) and Western blotting (right lane). (C) Copurification of NisB^E^_flag_ with LP_His_. Left lane, SDS-PAGE. Right lane, Western blotting. LP_His_, LP was C-terminally tagged by a 6×His tag. NisB_flag_ size, 120.2 kDa. NisB^G^_flag_ size, 86.5 kDa. NisB^E^_flag_ size, 36.6 kDa. NisA_His_ size, 7.2 kDa. LP_His_, 3.3 kDa. (D) Mechanistic possibilities for NisA-NisB interactions during catalysis. Model 1, leader peptide binds at a single site, which is located in the glutamylation domain of NisB throughout catalysis. Model 2, leader peptide binds different sites for glutamylation and elimination reaction. Only one monomer of the NisB dimer is shown. NisB is indicated in yellow. The leader peptide and core peptide are shown in red and purple, respectively. The leader peptide binding site in the glutamylation domain is indicated in blue, while the leader peptide binding site in the elimination domain is shown in green.

To evaluate whether the split NisBs are functional, the glutamylation domain (NisB^G^) and the elimination domain (NisB^E^) were separated by putting them on two different expression plasmids (Fig. 5B), one of which also encoded C-terminally 6×His-tagged NisA (NisA_His_), where the serine-containing linker was not introduced. The peptide was purified from the cytoplasm by affinity purification (Fig. 6A and B). MALDI-TOF MS analysis showed that NisA_His_ was fully modified with nine dehydrations by not only full-length NisB but also NisB^G^ and NisB^E^ together (Fig. 5C). These two separated NisB domains functioned equally as well as full-length NisB, although they were no longer covalently connected. As described above, nisin produced in the producing organism L. lactis is commonly dehydrated eight times, with Ser29 in the core peptide escaping dehydration (11). The observation of the ninth dehydration can be explained because NisC was absent, precluding ring formation, particularly for ring E. When NisB^E^ was not expressed and only NisB^G^ was present, the peptide intermediates in the dehydration process, i.e., a glutamylated peptide with two or three 129-Da adducts, were obtained (Fig. 5C), like the observed glutamylated His_6_-NisA in the *in vitro* reaction catalyzed by NisB with mutations in the elimination domain (11). According to these observations, we conclude that artificially divided NisB^G^ and NisB^E^ can perform the dehydration reaction in the core peptide of NisA with full functionality. In fact, the naturally split LanB-like dehydratases, TpdB (homologous to the glutamylation domain) and TpdC (homologous to the elimination domain), have been revealed in the biosynthesis of thiomuracin 11 and goadsporin 14 (39, 40). Together with this, our finding explains a consequence of dehydratase evolution in lanthipeptide biosynthesis. Additionally, we observed that only two or three Thr/Ser residues of the core peptide were glutamylated in the absence of NisB^E^, while glutamylation did occur. This implies that the glutamylation and elimination reactions alternate, as described in reactions catalyzed by TpdB and TpdC (39, 40). The elimination of the glutamyl residues initially formed is required for the next glutamylation step to occur.

### A potential leader peptide binding element in the elimination domain of NisB.

To characterize the interplay of NisA with the glutamylation domain and the elimination domain of NisB, pulldown assays with the purification of NisA_His_ were conducted. Consistent with previous studies (28, 32), abundant NisB was copurified with NisA_His_ when coexpressed in L. lactis (Fig. 6A and B). After deletion of the elimination domain in NisB, as expected, NisB^G^ in complex with NisA_His_ was isolated, since the NisB N-terminal region contains an embedded subdomain implicated by the structure of the NisAB complex (28), termed the RiPP recognition element (RRE) (41), which is essential for binding to the leader peptide (LP) of NisA prior to modification of the core peptide (CP). Interestingly, when the production of independent NisB^E^ was reintroduced, we found that not only NisB^G^ but also NisB^E^ was pulled down with NisA_His_. This seems to conflict with the LP-NisB interaction mechanism, where NisB uses a single LP binding site, the RRE motif located in the glutamylation domain, for glutamylation and elimination reactions (30) (Fig. 6D, model 1). One possible explanation is that the glutamylated CP of NisA_His_ that is ready for the elimination reaction binds to NisB^E^, resulting in the isolation of the NisA_His_-NisB^E^ complex, as the LP of NisA is essential for the glutamylation but not for the elimination reaction according to a previous study (28). To gain further insight, NisA_His_ and NisB^E^ were coexpressed in the absence of NisB^G^ to avoid the glutamylation reaction in the CP. Strikingly, NisB^E^ was still pulled down with unmodified NisA_His_, as shown by Western blotting (Fig. 6B). Furthermore, even without the presence of the CP, the 6×His-tagged LP (LP_His_) was found to be purified in complex with NisB^E^ (Fig. 6C). Taken together, we propose that the elimination domain of NisB contains an unknown leader peptide binding site that facilitates the elimination of glutamate on the core peptide, despite the notion that the LP seems not to be necessary for the elimination reaction performed *in vitro*. Therefore, our data argue against model 1 and support model 2, where the LP binds to different sites for glutamylation and elimination reactions (Fig. 6D). The LP of NisA initially binds to RRE, located in the N-terminal NisB, to ensure the CP is appropriately positioned in the active site of the glutamylation domain. After one or a few Thr/Ser residues in the CP are glutamylated, the LP dissociates with RRE and reassociates with another binding site located in the C-terminal NisB to position the glutamylated CP in the active site of the elimination domain, promoting the elimination reaction. Once the elimination reaction is completed, the next glutamylation reaction takes place again.

### Split NisBs allow subsequent efficient cyclization and transport of NisA.

**FIG 7:**
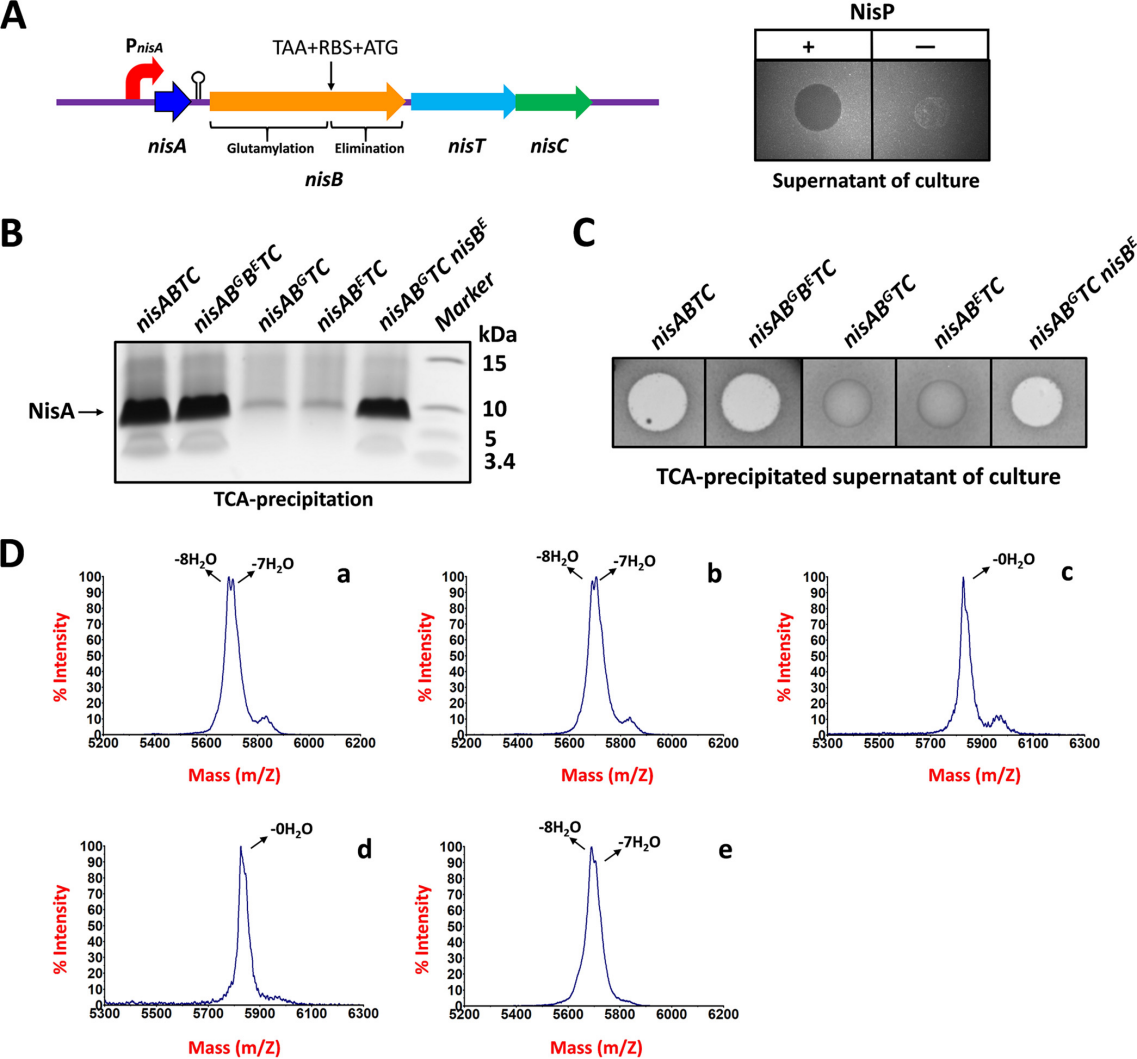
Split NisBs allow subsequent cyclization and transport. (A) NisB was split by inserting TAA-RBS-ATG between the glutamylation domain and the elimination domain. The left panel shows the gene operon *nisABTC* for nisin biosynthesis. The right panel displays antimicrobial activity. The supernatant of cell culture was incubated with purified NisP to remove the leader peptide. Indicator strain, Micrococcus flavus. (B) 16% Tricine SDS-PAGE for secreted NisA. The supernatant of cell culture was concentrated by TCA precipitation. (C) Antimicrobial activity assay for secreted NisA. The TCA-precipitated supernatant was incubated with purified NisP to remove the leader peptide. Indicator strain, Micrococcus flavus. (D) MALDI-TOF MS analysis of NisA in the TCA-precipitated supernatant. a, NZ9000/pTLR4-*nisABTC*. b, NZ9000/pTLR4-*nisAB^G^B^E^TC*. c, NZ9000/pTLR4-*nisAB^G^TC*. d, NZ9000/pTLR4-*nisAB^E^TC*. e, NZ9000/pTLR4-*nisAB^G^TC* pIL3-*nisB^E^*.

We have shown that split NisBs can efficiently dehydrate NisA equally as well as full-length NisB. However, the influence of NisB split on the following cyclization and transport for NisA remains to be evaluated. Initially, the stop codon TAA, a ribosome binding site, and the start codon ATG were inserted in the *nisB* gene between the glutamylation and elimination domain included in the wild-type operon *nisABTC* that is located in a multicopy expression plasmid, generating two independent genes corresponding to NisB domains (Fig. 7A). The supernatant of the cell culture displayed antimicrobial activity after incubation with the purified protease NisP (Fig. 7A), indicating that modified NisA with thioether rings was secreted outside the cells in spite of the split of NisB. To confirm this further, the supernatant was precipitated by use of trichloroacetic acid (TCA). SDS-PAGE showed that split NisBs gave rise to a good yield of secreted NisA, comparable to full-length NisB (Fig. 7B). Peptides from both concentrated supernatants exhibited identical antimicrobial activity (Fig. 7C) and were found to be dehydrated seven or eight times (Fig. 7D). In contrast, when the elimination domain or the glutamylation domain was deficient, the secretion efficiency of NisA was largely decreased (Fig. 7B). Antimicrobial activity was not detected for either TCA-precipitated supernatant (Fig. 7C), in line with the MALDI-TOF MS analysis that the secreted NisA in the supernatants was not dehydrated at all (Fig. 7D). However, when NisB^E^ encoded by another expression plasmid was introduced in the strain producing NisB^G^, a high-yield, good antimicrobial activity and full dehydration of the secreted peptide were retained (Fig. 7B to D). In summary, these results indicate that the split of NisB at the boundary between the glutamylation and elimination domains neither prevents the dehydration of NisA nor affects the subsequent cyclization by NisC and the peptide transport by NisT. Importantly, the presence of both NisB domains is required for NisT to transport NisA with full ability. It is expected that NisA that had not been modified was secreted by the strain missing the glutamylation domain, as the formation of glutamylated Thr/Ser is a prerequisite for the elimination reaction combined with dehydration. In the absence of the elimination domain, the glutamylated peptide actually accumulated within cells, as shown above (Fig. 5C), but it was not detected in the supernatant of the cell culture. Only the peptide without any modification was exported outside the cells. It is likely that the enlarged size of the core peptide, due to the glutamate adduct, blocks its transport through the channel created by NisT, thereby determining the substrate specificity of NisT during peptide transport.

### Both split NisB domains interact with NisC and NisT.

**FIG 8:**
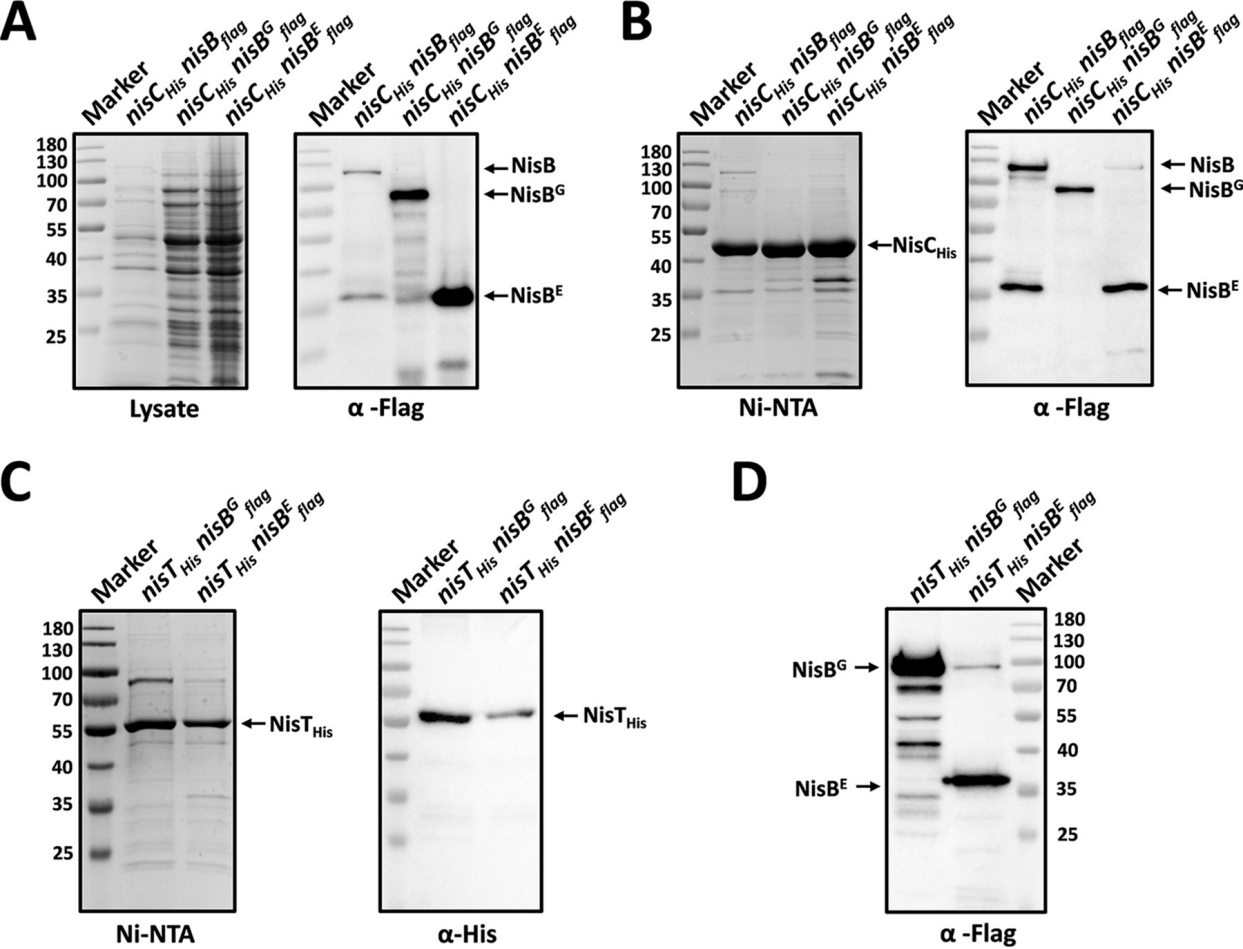
Interactions of split NisBs with NisC and NisT. (A and B) NisC_His_ was coexpressed with NisB_flag_, NisB^G^_flag_, or NisB^E^_flag_ in L. lactis NZ9000. (C and D) NisT_His_ was coexpressed with NisB^G^_flag_ or NisB^E^_flag_ in L. lactis NZ9000. The proteins were purified from the cell lysate by affinity purification using Ni-NTA agarose. The elutions were analyzed by 8% glycine SDS-PAGE followed by Coomassie G-250 staining, or Western blotting using anti-His and anti-Flag antibodies as indicated. NisT_His_ size, 70 kDa. NisC_His_ size, 48.7 kDa. NisB_flag_ size, 120.2 kDa. NisB^G^_flag_ size, 86.5 kDa. NisB^E^_flag_ size, 36.6 kDa.

NisB has been shown to interact with NisC and NisT to assemble a multimeric protein complex. It is interesting to know whether the interactions are maintained when NisB is split. For this reason, 6×His-tagged NisC was coexpressed with either NisB^G^ or NisB^E^ (Fig. 8A). SDS-PAGE and Western blotting indicated that both separated NisB domains were copurified with NisC_His_ (Fig. 8B). When NisT C-terminally tagged by 6×His tag (NisT_His_) was produced with coexpression of NisB^G^ or NisB^E^, the purification of NisT_His_ pulled down both independent NisB domains (Fig. 8C and D). Therefore, the interactions of both NisB domains with NisC and NisT were detected, providing detailed information about the architecture of the nisin biosynthesis machinery and emphasizing the importance of the association of both glutamylation and elimination domains of NisB with NisC and NisT for nisin maturation and transport. Additionally, we found that the size of the degraded C-terminal NisB copurified with NisC_His_ was the same as the size of NisB^E^ from Western blot analysis (Fig. 8B). This confirmed the hypothesis that cellular NisB is partially divided into two products at the boundary between the glutamylation domain and the elimination domain. In spite of NisB degradation, the modification and transport of nisin would not be affected due to the full functionality of split NisBs as described above.

## DISCUSSION

Lanthipeptides are synthesized by a putative membrane-associated lanthionine synthetase complex. The dehydration, cyclization, and transport of lanthipeptides have been well studied independently. However, information on the complete biosynthesis machinery for lanthipeptide modification and secretion is limited. In this study, the intact nisin biosynthesis complex NisBTC was, for the first time, isolated successfully, which was performed using a pulldown assay by isolating 6×His-tagged NisT from the cytoplasmic membrane of L. lactis, overexpressing nisin biosynthesis-related proteins. This provides direct evidence for the existence of such a complex within the cell membrane of the nisin producer. NisT was purified in abundance using the detergent DDM, and its band was clearly observed by SDS-PAGE. However, the copurified NisB and NisC were only detected by Western blotting, underlining a relatively weak affinity of both enzymes with the transporter. This is in accordance with the hypothesis that the multimeric lanthionine synthetase complex for nisin production is highly unstable and transient in nature (7). The biosynthesis processes of nisin, such as dehydration, cyclization, and secretion, were dissected *in vivo*. It has been demonstrated that NisB alone is capable of performing dehydration reactions entirely independently of the lanthionine synthetase complex (11, 28, 42). *In vitro* activity of NisC has been conducted in the absence of NisB and NisT (13). The dedicated nisin ABC transporter NisT can transport unmodified and dehydrated peptides without the presence of either NisB or NisC (15). Moreover, in cells efficiently secreting nisin, the fluorescently labeled nisin biosynthetic machinery could not be visualized directly. However, once the secretion pathway of nisin was blocked, the aggregation of the machinery was observed at the cell poles of L. lactis (4). Taken together, the NisBTC complex is not a prerequisite for correct functioning of any of the modification enzymes and the transporter, and its assembly *in vivo* seems to be a highly dynamic process. Besides the intact NisBTC complex probably carrying NisA, its NisT-associated subcomplexes, such as NisTB, NisTC, and NisBTC, could also be isolated from the cell membrane in the absence of NisA, as shown by pulldown assays. From this, a specific interaction between NisT and NisC was observed, in agreement with the previously determined interaction of NisT with NisC, by coimmunoprecipitation and a yeast two-hybrid assay (23). We also detected an interaction of NisT with NisB, which was not observed in the above-mentioned study, but in that case for SpaT and SpaB (26). Furthermore, the substrate peptide is not required for the association of the transporter with the modification machinery. NisBC likely plays a role as a courier to ship fully modified NisA to NisT for transport beyond the function of modification. In contrast, NisT is inaccessible to unmodified NisA that is dissociated from NisBC, which is likely the reason for the extremely low secretion efficiency by NisT when NisB and NisC were deficient (37). Therefore, the direct interaction between NisB and/or NisC and NisT not only implies a crucial role for NisBC in the highly efficient secretion of NisA but also suggests a mechanism by which the premature secretion of unfinished NisA is prevented. Our data indicate that NisB possesses a stronger affinity to NisT than NisC. A previous study reported that the secretion of NisA was nearly completely abolished by deficiency of NisB (37). Single-cell analysis showed that NisB can recruit NisT, which is homogeneously distributed within the cytoplasmic membrane to the cell poles (4). Overall, it is commonly accepted that NisB is a main component of the NisBTC complex, which is necessary for the delivery of the modified NisA to NisT, suggesting a channeling mechanism of NisA transfer between the nisin modification enzymes and the transporter (24, 37).

Prior to transport, NisA is modified by the maturation machinery NisBC. The assembly of the NisABC complex has been demonstrated *in vitro* (35). In the cells of the strain where NisA, NisB, and NisC were overexpressed, our analysis showed the cellular abundance of nisin modification-related protein complexes with high concentrations of NisAB/NisAC and a low concentration of NisABC. This suggests a transient assembly of NisABC via an alternating binding mechanism to achieve nisin modification. NisA is first modified by NisB. NisB in complex with NisA encounters NisC, transfers dehydrated NisA to NisC, and disassociates with NisC as soon as the leader peptide binds to NisC. NisC cyclizes newly formed dehydrated residues to free cysteines. Subsequently, NisC in complex with NisA encounters NisB, transfers NisA with newly formed rings to NisB, and dissociates from NisB once the leader peptide binds to NisB again. This process goes on in consecutive steps until NisA is fully modified, which is supported by the proposed alternating actions of NisB and NisC, whose activities follow one after another in a repetitive way (38). An *in vitro* experiment reported that NisBC is formed only when the substrate NisA is present (35). In contrast, we found that *in vivo* NisB can interact with NisC directly. Through the direct specific interaction that promotes the transfer of NisA to each other, the coordinated and alternating modification manners are therefore conducted. We observed that the NisAC complex is more stable than NisAB, as NisA was still partially in complex with NisC but totally released from NisB after heat treatment, and NisB was largely removed from NisA by high-concentration imidazole, but NisC was not, in a purification process, which also indicates a stronger affinity of NisA to NisC than that to NisB. This is not in agreement with the study that the interaction of NisA with NisB appears to be the strongest, since only a small amount of NisC was copurifed with engineered NisA (32). However, the low yield of copurified NisC was likely caused by its lower cellular expression level compared to NisB due to the location of their genes in the *nisBTC* operon. In previous studies, the interactions of NisA with both NisB and NisC have been investigated *in vitro* by performing quantitation experiments. Dehydrated residues appear to increase the affinity of NisA for NisB, whereas thioether rings reduce the affinity (33). On the contrary, NisC binds NisA in different modification states with similar affinities (34). According to these two studies, NisC possesses a five times higher affinity for the modified NisA than NisB. This is consistent with our findings described above. Furthermore, the analyses for the NisA variants with different numbers of cysteine residues in the core peptide suggest a tendency for NisC to lose affinity to NisA with an increasing number of completed lanthionine rings. Hence, it is tempting to speculate that NisA containing all five finished rings is associated with NisBC mainly via NisC and is exported outside the cells by NisT after release from the NisBC complex due to the decreased affinity of NisC to fully modified NisA, once NisBC is targeted to NisT. Thus, unmodified NisA and various precursor nisin intermediates, especially dehydrated NisA without rings, will be prevented from being secreted prematurely.

Among the nisin biosynthesis-related proteins, good structural information of NisB and NisC has been reported (28, 43). NisB is a dimer with each monomer composed of one glutamylation domain and one elimination domain. We found that a split of NisB between these two domains still enables NisA to be fully dehydrated, indicating a strong plasticity of NisB. The data suggest that each separated domain performs their corresponding reactions in an alternating manner and independently of the covalent connection between them. Based on the orientation of the glutamylation and elimination domains in NisB and MibB, it has been proposed that dimerization of lanthipeptide dehydratases is a requirement for catalysis (44). Distance constraints also suggest that glutamylation of the core peptide occurs within the glutamylation domain of one monomer, while glutamate elimination occurs within the elimination domain of the other monomer (44). In our study, when NisB was split, the majority of NisB^G^ was purified as a monomer in solution (Fig. S7A and B), while the question of whether NisB^E^ is a monomer or maintained its original dimer state could not be answered. The monomeric state of functional NisB^G^ indicates that the dimerization of the glutamylation domain is unnecessary for its reaction. To understand the alternating manner of NisB^G^ and NisB^E^ in detail, how NisA is transferred between split NisBs and whether they directly interact with each other remains to be elucidated. NisB is not very stable in cells, as two natural degradation products, a large N-terminal NisB and a small C-terminal NisB, were frequently found in the cytoplasm and at the cell membrane of nisin-producing strains previously (22, 32, 33). Our data indicate that the degraded products are exactly the separated glutamylation domain and elimination domain. Despite degradation of NisB in the cells, the biosynthesis and secretion of fully modified NisA would not be negatively affected, because split NisBs are functional, as well as the full-length NisB, and allow the following full cyclization and subsequent efficient transport. Actually, naturally split LanBs (TpdB/TpdC) have been found and characterized: TbtB/TbtC from Thermobispora bispora, which are involved in the synthesis of thiomuracin 11 (39), and GodF/GodG from *Streptomyces* sp. strain TP-A0584, which are involved in the synthesis of goadsporin 14 (40). Both TbtB and GodF have been shown to catalyze the glutamylation of dehydratable serine residues. In thiomuracin 11 biosynthesis, the glutamylation reaction has been shown to involve Glu-tRNA^Glu^, originating from the aminoacyl-tRNA pool of the cell, as for LanBs (39). Most of the residues identified as forming the active site of the glutamylation domain and all the residues involved in the elimination step in NisB are conserved in split LanBs. The TbtB structure shows that it is present in the crystal as a monomer (29), similar to NisB^G^, as we described above. The glutamylation and elimination reactions catalyzed by split LanBs also alternate (45). Thus, the dehydration reaction catalyzed by the two-domain full-length LanBs or two single-domain split LanB enzymes, TpdBs and TpdCs, is performed in a similar manner, which suggests a divergent evolution within the LanB family.

10.1128/mBio.02585-21.7 FIG S7 Purification and analysis of NisB^G^_His_. NisB^G^_His_ and NisB^E^_His_ were expressed in L. lactis NZ9000. The proteins were purified from the cell lysate by affinity purification using Ni-NTA agarose. (A) SEC analysis of the NisB^G^_His_ elution after affinity purification. The SEC column ENrich SEC 650 10/300 was calibrated using gel filtration standard (red dotted peaks), which is composed of thyroglobulin (670 kDa), ϒ-globulin (158 kDa), ovalbumin (44 kDa), myoglobin (17 kDa), and vitamin B_12_ (13.5 kDa), at 4°C while measuring absorbance at 280 nm (mAU) per ml elution volume. The eluted peaks are indicated with the state of NisB^G^ oligomerization. (B) Elution fractions of NisB^G^_His_ by SEC were analyzed by 8% glycine SDS-PAGE. Red box represents the SEC fractions containing monomeric NisB^G^_His_. NisB^G^_His_, the glutamylation domain of NisB C-terminally labelled by a 6×His tag. NisB^E^_His_, the elimination domain of NisB C-terminally labelled by a 6×His tag. NisB^G^_His_ size, 84.6 kDa. NisB^E^_His_ size, 34.7 kDa. Download FIG S7, TIF file, 1.2 MB. Copyright © 2021 Chen and Kuipers. 2021 Chen and Kuipers. https://creativecommons.org/licenses/by/4.0/ This content is distributed under the terms of the Creative Commons Attribution 4.0 International license.

The presence of a leader peptide is essential for precursor peptide interaction with LanBs. In particular, the conserved FNLD box within the leader peptide of NisA has been shown to be a key element for substrate recognition (32, 33, 46). Consistent with this finding, determination of the structure of the NisAB complex implicated this motif in a strong network of hydrophobic interactions with the peptide leader binding domain, also termed RRE, of NisB, which is located in the glutamylation domain (28). Ortega et al. studied the involvement of the leader peptide in the glutamylation and elimination activities of NisB separately. They found that the leader peptide of NisA was essential for glutamylation but not for elimination, which suggests that the local structure for glutamylated nisin is sufficient for binding and processing by the elimination domain (28). Moreover, Repka et al. engineered a disulfide that covalently links the leader peptide of NisA to the RRE of NisB, confirming the functional leader peptide binding site and supporting a mechanism where NisB uses a single leader peptide binding site for glutamylation and elimination (30). However, in our study, the binding of glutamylated NisA, unmodified NisA, and even independent leader peptide to the separated elimination domain of NisB was detected, implying a specific interaction of the leader peptide with the elimination domain of NisB besides the proposed direct recognition of glutamylated nisin by the elimination domain. It is likely that an unknown recognition site is present in this domain for leader peptide binding. Hence, this finding supports a model where the leader peptide binds to different sites, which are located in corresponding domains for glutamylation and elimination, arguing with the model mentioned above. Additionally, the observation of the specific interactions of the elimination domain of NisB with NisC and NisT suggests that NisA, when glutamate elimination has been finished, is transferred to NisC or NisT from the elimination domain for the desired cyclization or transport. Future experiments to elucidate the detailed molecular interaction of the leader peptide with the elimination domain of NisB will give in-depth insights into the dehydration mechanism by NisB.

In conclusion, we present direct evidence for the existence of the nisin biosynthesis machinery in the cytoplasmic membrane when the secretion of precursor nisin is either available or blocked. By analyses of the interactions within the intact NisBTC complex and the modification machinery NisABC, their cooperative actions for the modification and transport of nisin were elucidated. The functional characterization of split NisBs *in vivo* provides an evolutionary clue of the LanB family for lanthipeptide dehydration. Importantly, based on the interaction of the leader peptide of NisA with the elimination domain of NisB, we speculate that a potential leader peptide binding domain is present in the C-terminal part of NisB, supporting a model where the leader peptide binds to different sites for glutamylation and elimination.

## MATERIALS AND METHODS

### Bacterial strains and growth conditions.

Bacterial strains of E. coli and L. lactis employed in this study are listed in Table S1 in the supplemental material. The bacterial strain L. lactis NZ9700 was used as the source of nisin biosynthetic genes. Micrococcus flavus was used as the indicator strain for the production of modified nisin. E. coli DH5α served as a host for cloning and plasmid preparation and was grown in Luria-Bertani (LB) medium at 37°C under aerobic conditions. L. lactis NZ9000 was used as an expression system and grown as a standing culture at 30°C in Difco M17 medium (BD, Franklin Lakes, NJ, USA) with 0.5% (wt/vol) glucose (GM17) or minimal essential medium (MEM) with 0.5% (wt/vol) glucose. The antibiotics were added when necessary at 100 μg/ml ampicillin for E. coli and 5 μg/ml chloramphenicol and/or 5 μg/ml erythromycin for L. lactis. For induction in L. lactis, 10 ng/ml nisin Z was added to the medium to initiate the expression of genes under the control of the nisin-inducible promoter P*_nisA_*, when the optical density at 600 nm (OD_600_) of cell culture reached to 0.6; 1.5% (wt/vol) agar was added to the growth medium as solid medium. All chemicals were purchased from Sigma-Aldrich.

10.1128/mBio.02585-21.8 TABLE S1 Strains used in this study. Download Table S1, DOCX file, 0.02 MB. Copyright © 2021 Chen and Kuipers. 2021 Chen and Kuipers. https://creativecommons.org/licenses/by/4.0/ This content is distributed under the terms of the Creative Commons Attribution 4.0 International license.

### Recombinant DNA techniques and oligonucleotides.

Plasmids used and constructed in this study are listed in Table S2. The techniques of standard molecular cloning were performed as described previously (47). The GenElute genomic DNA kit (Sigma-Aldrich, St. Louis, MO) was used to isolate genomic DNA of L. lactis. The NucleoSpin Plasmid EasyPure kit (Bioke, Leiden, the Netherlands) and the NucleoSpin gel & PCR clean-up kit (Bioke, Leiden, the Netherlands) were employed to extract plasmids and purify PCR products by following the manufacturer’s instructions. PCRs were conducted with PrimeSTAR Max DNA polymerase (TaKaRa Bio Europe SAS, Saint-Germain-en-Laye, France) according to the manufacturer’s protocol. The obtained PCR products were mixed and treated with the Gibson assembly master mix (Bioke, Leiden, the Netherlands), yielding 20-nucleotide overhangs annealing to complementary overhangs. The mixtures were applied to transform E. coli DH5α directly or L. lactis NZ9000 after desalting to generate plasmids. The transformation of E. coli strains was performed by heat shock by following standard procedures (47). Electrocompetent cells of L. lactis were transformed using electroporation with a Bio-Rad gene Pulser (Bio-Rad Laboratories, Richmond, CA) (48). All nucleotide sequencing was performed at Macrogen Europe (Amsterdam, the Netherlands). Oligonucleotides used in this work were purchased from Biolegio BV (Nijmegen, the Netherlands) and are given in Table S3.

10.1128/mBio.02585-21.9 TABLE S2 Plasmids used in this study. Download Table S2, DOCX file, 0.02 MB. Copyright © 2021 Chen and Kuipers. 2021 Chen and Kuipers. https://creativecommons.org/licenses/by/4.0/ This content is distributed under the terms of the Creative Commons Attribution 4.0 International license.

10.1128/mBio.02585-21.10 TABLE S3 Oligonucleotides used in this study. Download Table S3, DOCX file, 0.01 MB. Copyright © 2021 Chen and Kuipers. 2021 Chen and Kuipers. https://creativecommons.org/licenses/by/4.0/ This content is distributed under the terms of the Creative Commons Attribution 4.0 International license.

### Trichloroacetic acid precipitation.

L. lactis was grown in minimal essential medium with the induction of 10 ng/ml nisin Z. Trichloroacetic acid (TCA; 100% [wt/vol]) was added to 45 ml supernatant of a cell culture with a final concentration of 10% (wt/vol) TCA. The mixture was kept on ice for 2 h and then centrifuged at 10,000 × *g* for 60 min at 4°C. The pellet was retained after discarding the supernatant. Subsequently, half the original volume of iced acetone was added to the pellet. After 60 min of centrifugation at 10,000 × *g* again, the pellet was retained and dried by vacuum-freezing desiccation. Finally, the dry pellet was resuspended in 0.5 ml 50 mM Tris-HCl, pH 7.0.

### Antimicrobial activity assay.

Micrococcus flavus was used as the indicator strain and grown overnight in GM17 under aerobic conditions. Diluted culture (100 μl; OD_600_, 0.5) was added to 100 ml melted GM17-agar at 45°C and poured in plates. A 10-μl sample with the addition of 1 μl purified protease NisP (Lab stock) was dropped on the plate after the agar was solid. The plates were left overnight at 30°C.

### Mass spectrometric analysis.

A volume of 1 μl of each sample was spotted, dried, and washed with Milli-Q water on the target. Subsequently, 1 μl of 5 mg/ml α-cyano-4-hydroxycinnamic acid (Sigma-Aldrich) was spotted on top of the samples. An ABI Voyager DE Pro (Applied Biosystems) MALDI-TOF operating in linear mode using external calibration was used to obtain mass spectra.

### Peptide and cellular protein purification.

L. lactis was grown overnight in GM17 medium with appropriate antibiotics. The overnight culture was 5% diluted in fresh GM17 medium and grown at 30°C. When the OD_600_ of cell culture was increased to 0.6, nisin Z was added to induce protein expression with a final concentration of 10 ng/ml. After 3 h of growth, cells were collected by centrifugation and washed twice with 50 mM Tris-HCl, pH 7.4. The harvested cells were resuspended in lysis buffer (50 mM NaH_2_PO_4_, 300 mM NaCl, and 10 mM imidazole, pH 8.0) with 10 mg/ml lysozyme and protease inhibitor and were incubated for 60 min at 37°C. MgSO_4_ (10 mM) and 100 mg/ml DNase I were added. After incubation for 5 min at 37°C, the suspension passed three times through a cell disruptor machine. Two centrifugation steps at 13,000 × *g* for 10 min at 4°C were performed to remove cell debris, and the cell lysate was obtained. For Ni-NTA purification, a standard procedure was followed and conducted in a cold room (4°C). A volume of 5 ml lysis buffer was run over the column containing Ni-NTA agarose (50%, 1.0 ml; Qiagen Benelux B.V.) to equilibrate it. Subsequently, 10 ml of the lysate flowed through the column material twice to allow 6×His-tagged peptide/protein to bind to the Ni-NTA agarose. Next, the column material was washed twice with 10 ml wash buffer (50 mM NaH_2_PO_4_, 300 mM NaCl, and 20 mM imidazole, pH 8.0). Elutions were collected in 3 fractions (250 μl, 750 μl, and 500 μl) using elution buffer (50 mM NaH_2_PO_4_, 300 mM NaCl, and 250 mM imidazole, pH 8.0). When required, proteins were further purified by using size-exclusion chromatography (SEC; ENrich SEC 650 10/300 column). Finally, purified peptides and proteins were analyzed by SDS-PAGE and Western blotting. For the removal of the linker sequence and the 6×His tag from the peptides, commercial factor Xa protease was used.

### Membrane protein purification.

The expression of membrane proteins was conducted as described above. Cells were collected by centrifugation and washed twice with 50 mM Tris-HCl, pH 7.4. The harvested cells were resuspended in lysis buffer (50 mM Tris-HCl and 300 mM KCl, pH 7.4) with 10 mg/ml lysozyme and protease inhibitor and were incubated for 60 min at 37°C. MgSO_4_ (10 mM) and 100 mg/ml DNase I were added. After incubation for 5 min at 37°C, the suspension was passed three times through a cell disruptor machine. Membrane fractions were obtained by ultracentrifugation and resuspended in binding buffer (50 mM Tris-HCl, 300 mM NaCl, and 10 mM imidazole, pH 7.4). The total membrane protein concentration was measured by bicinchoninic acid (BCA) assay (Thermo Fischer Scientific). Membranes were solubilized with 1% (wt/vol) *n*-dodecyl-β-d-maltoside (DDM) for 2 h at 4°C. Insoluble material was removed by ultracentrifugation at 40,000 × g for 30 min. A volume of 10 ml binding buffer with 0.1% (wt/vol) DDM was run over the column containing Ni-NTA agarose (50%, 1.0 ml; Qiagen Benelux B.V.) to equilibrate it. Subsequently, 10 ml of the soluble membrane was mixed with 0.5 ml Ni-NTA agarose and incubated at 4°C for 2 h with shaking to allow 6×His-tagged protein to bind to the Ni-NTA agarose. The soluble membrane flowed through the column material. Next, the column material was washed twice with 10 ml wash buffer (50 mM Tris-HCl, 300 mM NaCl, 20 to ∼35 mM imidazole, and 0.1% [wt/vol] DDM, pH 7.4). Elutions were collected in 3 fractions (250 μl, 750 μl, and 500 μl) using elution buffer (50 mM Tris-HCl, 300 mM NaCl, 300 mM imidazole, and 0.1% [wt/vol] DDM, pH 7.4). Finally, purified membrane proteins were analyzed by SDS-PAGE and Western blotting.

### Cell fractionation.

The cytoplasm and membrane fractions were separated: the cell pellet was washed with 50 mM Tris-HCl, pH 7.4, resuspended in cell lysis buffer, and disrupted by a cell disruptor machine. The obtained lysate was centrifuged to remove cell debris. The supernatant was then ultracentrifuged (40,000 × *g* for 1 h, 4°C), and the new supernatant (cytoplasmic fraction) was collected again. The membrane pellet was resuspended in cell lysis buffer and ultracentrifuged again (40,000 × *g* for 30 min, 4°C). Finally, the collected membrane fraction was resuspended in the lysis buffer. BCA reagent was used to determine the protein concentrations of all collected fractions, and 30 μg total protein was loaded per lane when SDS-PAGE was performed.

### SDS-PAGE and Western blotting.

The samples for glycine or tricine SDS-PAGE were incubated in loading buffer containing 5% (vol/vol) β-mercaptoethanol and boiled for 10 min. SDS-PAGE was performed according to a standard operation manual (47). Western blots were performed using anti-His, anti-leader, anti-Flag, anti-NisC, and anti-green fluorescent protein antibodies.
