# Supplementary material for: Isolation and Analysis of the Nisin Biosynthesis Complex NisBTC: further Insights into Their Cooperative Action
Source: mBio. 2021 Oct 5;12(5):e02585-21. doi: 10.1128/mBio.02585-21 (PMC8546558; doi:10.1128/mBio.02585-21)
Supplement: TABLE S1 [file mbio.02585-21-st001.docx]

**Table S1 Strains used in this study**

| **Strains** | **Genotype** | **Source** |
| --- | --- | --- |
| *L. lactis* NZ9700 | *nisABTCIPRKFEG*, nisin producer | ([1](#_ENREF_1)) |
| *E. coli* DH5α | F^-^∆*lac*U169(Ø80d *lac*Z∆M15) *sup*E44 *hsd*R17 *rec*A1 *gyr*A96 *end*A1 *thi*-1 *rel*A1 | ([2](#_ENREF_2)) |
| *Micrococcus flavus* | Indicator strain for nisin | ([3](#_ENREF_3)) |
| *L. lactis* NZ9000 | MG1363 *pepN::nisRK* | Lab stock |
| SQ01 | NZ9000 pNZE3-*nisAT*, pIL3-*nisBC*; ery^r^, cm^r^ | This study |
| SQ02 | NZ9000 pNZE3-*nisAT*, pIL3-*nisB_flag_C*; ery^r^, cm^r^ | This study |
| SQ03 | NZ9000 pNZE3-*nisAT_His_*, pIL3-*nisB_flag_C*; ery^r^, cm^r^ | This study |
| SQ04 | NZ9000 pNZE3-*nisAT*^H551A^*_His_*, pIL3-*nisB_flag_C*; ery^r^, cm^r^ | This study |
| SQ05 | NZ9000 pNZE3-*nisT*, pIL3-*nisB_flag_C*; ery^r^, cm^r^ | This study |
| SQ06 | NZ9000 pNZE3-*nisT_His_*, pIL3-*nisB_flag_C*; ery^r^, cm^r^ | This study |
| SQ07 | NZ9000 pNZE3-*nisAT_His_*; ery^r^ | This study |
| SQ08 | NZ9000 pNZE3-*nisT_His_*, pIL3-*nisB_flag_*; ery^r^, cm^r^ | This study |
| SQ09 | NZ9000 pNZE3-*nisT_His_*, pIL3-*nisC*; ery^r^, cm^r^ | This study |
| SQ10 | NZ9000 pNZE3-*nisAT^sfgfp^*, pIL3-*nisB_flag_C*; ery^r^, cm^r^ | This study |
| SQ11 | NZ9000 pNZE3-*nisAT^sfgfp^_His_*, pIL3-*nisB_flag_C*; ery^r^, cm^r^ | This study |
| SQ12 | NZ9000 pNZE3-*nisA_GS-His_*-*nisB_flag_*; ery^r^ | This study |
| SQ13 | NZ9000 pNZE3-*nisA_GS-His_*-*nisB_flag_*, pIL3-*nisC*; ery^r^, cm^r^ | This study |
| SQ14 | NZ9000 pNZE3-*nisA4_GS-His_*-*nisB_flag_*, pIL3-*nisC*; ery^r^, cm^r^; Peptide CCCCA | This study |
| SQ15 | NZ9000 pNZE3-*nisA3_GS-His_*-*nisB_flag_*, pIL3-*nisC*; ery^r^, cm^r^; Peptide CCCAA | This study |
| SQ16 | NZ9000 pNZE3-*nisA2_GS-His_*-*nisB_flag_*, pIL3-*nisC*; ery^r^, cm^r^; Peptide CCAAA | This study |
| SQ17 | NZ9000 pNZE3-*nisA1_GS-His_*-*nisB_flag_*, pIL3-*nisC*; ery^r^, cm^r^; Peptide CAAAA | This study |
| SQ18 | NZ9000 pNZE3-*nisA0_GS-His_*-*nisB_flag_*, pIL3-*nisC*; ery^r^, cm^r^; Peptide AAAAA | This study |
| SQ19 | NZ9000 pNZE3-*nisA_GS-His_*, pIL3-*nisC*; ery^r^, cm^r^ | This study |
| SQ20 | NZ9000 pIL3-*nisC_His_*; cm^r^ |  |
| SQ21 | NZ9000 pNZE3-*nisB_flag_*, pIL3-*nisC_His_*; ery^r^, cm^r^ | This study |
| SQ22 | NZ9000 pNZE3-*nisA_His_B*; ery^r^ | This study |
| SQ23 | NZ9000 pNZE3-*nisA_His_B^G^*; ery^r^ | This study |
| SQ24 | NZ9000 pNZE3-*nisA_His_B^G^*, pIL3-*nisB^E^*; ery^r^, cm^r^ | This study |
| SQ25 | NZ9000 pNZE3-*nisA_His_B_flag_*; ery^r^ | This study |
| SQ26 | NZ9000 pNZE3-*nisA_His_B^G^_flag_*; ery^r^ | This study |
| SQ27 | NZ9000 pNZE3-*nisA_His_*, pIL3-*nisB^E^_flag_*; ery^r^, cm^r^ | This study |
| SQ28 | NZ9000 pNZE3-*nisA_His_B^G^_flag_*, pIL3-*nisB^E^_flag_*; ery^r^, cm^r^ | This study |
| SQ29 | NZ9000 pNZE3-*LP_His_*, pIL3-*nisB^E^_flag_*; ery^r^, cm^r^; LP, leader peptide of NisA | This study |
| SQ30 | NZ9000 pTLR4-*nisABTC*; ery^r^ | This study |
| SQ31 | NZ9000 pTLR4-*nisAB^G^B^E^TC*; ery^r^ | This study |
| SQ32 | NZ9000 pTLR4-*nisAB^G^TC*; ery^r^ | This study |
| SQ33 | NZ9000 pTLR4-*nisAB^E^TC*; ery^r^ | This study |
| SQ34 | NZ9000 pTLR4-*nisAB^G^TC*, pIL3-*nisB^E^*; ery^r^, cm^r^ | This study |
| SQ35 | NZ9000 pNZE3-*nisB^G^_flag_*, pIL3-*nisC_His_*; ery^r^, cm^r^ | This study |
| SQ36 | NZ9000 pNZE3-*nisB^E^_flag_*, pIL3-*nisC_His_*; ery^r^, cm^r^ | This study |
| SQ37 | NZ9000 pNZE3-*nisB^G^_flag_*, pIL3-*nisT_His_*; ery^r^, cm^r^ | This study |
| SQ38 | NZ9000 pNZE3-*nisB^E^_flag_*, pIL3-*nisT_His_*; ery^r^, cm^r^ | This study |
| SQ39 | NZ9000 pNZE3-*nisB^G^_His_*; ery^r^ | This study |
| SQ40 | NZ9000 pNZE3-*nisB^E^_His_*; ery^r^ | This study |

**References**

1. de Ruyter PG KO, de Vos WM. 1996. Controlled gene expression systems for *Lactococcus lactis* with the food-grade inducer nisin. Appl Environ Microbiol 62:3662-3667.

2. Chen JQ, Zhao LQ, Fu G, Zhou WJ, Sun YX, Zheng P, Sun JB, Zhang DW. 2016. A novel strategy for protein production using non-classical secretion pathway in *Bacillus subtilis*. Microb Cell Fact 15:69.

3. van Heel AJ, Mu DD, Montalban-Lopez M, Hendriks D, Kuipers OP. 2013. Designing and producing modified, new-to-nature peptides with antimicrobial activity by use of a combination of various lantibiotic modification enzymes. ACS Synth Biol 2:397-404.
