# Supplementary material for: Isolation and Analysis of the Nisin Biosynthesis Complex NisBTC: further Insights into Their Cooperative Action
Source: mBio. 2021 Oct 5;12(5):e02585-21. doi: 10.1128/mBio.02585-21 (PMC8546558; doi:10.1128/mBio.02585-21)
Supplement: TABLE S2 [file mbio.02585-21-st002.docx]

**Table S2 Plasmids used in this study**

| **Plasmids** | **Characteristics** | **Source** |
| --- | --- | --- |
| pNZE3-*nisA* | P*_nisA_*, *nisA*; ery^r^ | Lab stock |
| pNZE3-*nisT* | P*_nisA_*, *nisT*; ery^r^ | This study |
| pNZE3-*nisAT* | P*_nisA_*, *nisA, nisT*; ery^r^ | This study |
| pNZE3-*nisAT_His_* | P*_nisA_*, *nisA, nisT_His_*; ery^r^; *nisT_His_*, NisT is C-terminally tagged by a 6xHis-tag | This study |
| pNZE3-*nisAT*^H551A^*_His_* | P*_nisA_*, *nisA, nisT*^H551A^*_His_*; ery^r^; *nisT*^H551A^*_His_*, *nisT_His_* with the mutation H551A | This study |
| pNZE3-*nisT_His_* | P*_nisA_*, *nisT_His_*; ery^r^; *nisT_His_*, NisT is C-terminally tagged by a 6xHis-tag | This study |
| pNZE3-*nisAT^sfgfp^* | P*_nisA_*, *nisA, nisT^sfgfp^*; ery^r^; *nisT^sfgfp^*, sfGFP is fused to the C-terminus of NisT | This study |
| pNZE3-*nisAT^sfgfg^_His_* | P*_nisA_*, *nisA, nisT^sfgfp^_His_*; ery^r^; *nisT^sfgfp^_His_*, a 6xHis-tag is fused to the C-terminus of the fusion protein NisT-sfGFP | This study |
| pNZE3-*nisA_GS-His_*-*nisB_flag_* | P*_nisA_*, *nisA_GS-His_*; ery^r^; *nisA_GS-His_*, NisA is C-terminally tagged by the factor Xa sequence, a linker, and a 6xHis-tag; *nisB_flag_*, NisB is C-terminally labelled by a Flag-tag | This study |
| pNZE3-*nisA4_GS-His_*-*nisB_flag_* | P*_nisA_*, *nisA4_GS-His_*, *nisB_flag_*; ery^r^; Peptide CCCCA | This study |
| pNZE3-*nisA3_GS-His_*-*nisB_flag_* | P*_nisA_*, *nisA3_GS-His_*, *nisB_flag_*; ery^r^; Peptide CCCAA | This study |
| pNZE3-*nisA2_GS-His_*-*nisB_flag_* | P*_nisA_*, *nisA2_GS-His_*, *nisB_flag_*; ery^r^; Peptide CCAAA | This study |
| pNZE3-*nisA1_GS-His_*-*nisB_flag_* | P*_nisA_*, *nisA1_GS-His_*, *nisB_flag_*; ery^r^; Peptide CAAAA | This study |
| pNZE3-*nisA0_GS-His_*-*nisB_flag_* | P*_nisA_*, *nisA0_GS-His_*, *nisB_flag_*; ery^r^; Peptide CAAAA | This study |
| pNZE3-*nisB_flag_* | P*_nisA_*, *nisB_flag_*; ery^r^;  *nisB_flag_*, NisB is C-terminally tagged by Flag-tag | This study |
| pNZE3-*nisA_His_B* | P*_nisA_*, *nisA_His_*, *nisB*; ery^r^; *nisA_His_*, NisA is C-terminally tagged by a 6xHis-tag | This study |
| pNZE3-*nisA_His_B^G^* | P*_nisA_*, *nisA_His_*, *nisB^G^*; ery^r^; *nisA_His_*, NisA is C-terminally tagged by a 6xHis-tag; *nisB^G^*, the glutamylation domain of NisB | This study |
| pNZE3-*nisA_His_B_flag_* | P*_nisA_*, *nisA_His_*, *nisB_flag_*; ery^r^; *nisA_His_*, NisA is C-terminally tagged by a 6xHis-tag; *nisB_flag_*, NisB is C-terminally tagged by Flag-tag | This study |
| pNZE3-*nisA_His_B^G^_flag_* | P*_nisA_*, *nisA_His_*, *nisB^G^_flag_*; ery^r^; *nisA_His_*, NisA is C-terminally tagged by a 6xHis-tag; *nisB^G^_flag_*, NisB^G^ is C-terminally tagged by Flag-tag | This study |
| pNZE3-*LP_His_* | P*_nisA_*, *LP_His_*; ery^r^; *LP_His_*, the leader peptide of NisA is C-terminally tagged by a 6xHis-tag | This study |
| pNZE3-*nisB^G^_flag_* | P*_nisA_*, *nisB^G^_flag_*; ery^r^;  *nisB^G^_flag_*, NisB^G^ is C-terminally tagged by Flag-tag | This study |
| pNZE3-*nisB^E^_flag_* | P*_nisA_*, *nisB^E^_flag_*; ery^r^;  *nisB^E^_flag_*, the elimination domain of NisB is C-terminally tagged by Flag-tag | This study |
| pIL3-*nisBTC* | P*_nisA_*, *nisBTC*; cm^r^ | Lab stock |
| pIL3-*nisBC* | P*_nisA_*, *nisBC*; cm^r^ | This study |
| pIL3-*nisB_flag_C* | P*_nisA_*, *nisB_flag_C*; cm^r^; NisB is C-terminally tagged by Flag-tag | This study |
| pIL3-*nisB_flag_* | P*_nisA_*, *nisB_flag_*; cm^r^; NisB is C-terminally tagged by Flag-tag | This study |
| pIL3-*nisC* | P*_nisA_*, *nisC*; cm^r^ | This study |
| pIL3-*nisC_His_* | P*_nisA_*, *nisC_His_*; cm^r^;  *nisC_His_*, NisC is C-terminally tagged by a 6xHis-tag | This study |
| pIL3-*nisB^E^* | P*_nisA_*, *nisB^E^*; cm^r^;  *nisB^E^*, the elimination domain of NisB | This study |
| pIL3-*nisB^E^_flag_* | P*_nisA_*, *nisB^E^_flag_*; cm^r^;  *nisB^E^_flag_*, NisB^E^ is C-terminally tagged by Flag-tag | This study |
| pIL3-*nisT_His_* | P*_nisA_*, *nisT_His_*; cm^r^;  *nisT_His_*, NisT is C-terminally tagged by a 6xHis-tag | This study |
| pTLR4-*nisABTC* | P*_nisA_*, *nisABTC*; ery^r^ | Lab stock |
| pTLR4-*nisAB^G^B^E^TC* | P*_nisA_*, *nisA*, *nisB^G^*, *nisB^E^*, *nisT*, *nisC*; ery^r^; *nisB^G^*, the glutamylation domain of NisB;  *nisB^E^*, the elimination domain of NisB; | This study |
| pTLR4-*nisAB^G^TC* | P*_nisA_*, *nisA*, *nisB^G^*, *nisT*, *nisC*; ery^r^; *nisB^G^*, the glutamylation domain of NisB | This study |
| pTLR4-*nisAB^E^TC* | P*_nisA_*, *nisA*, *nisB^E^*, *nisT*, *nisC*; ery^r^; *nisB^E^*, the elimination domain of NisB | This study |
