# Supplementary material for: Isolation and Analysis of the Nisin Biosynthesis Complex NisBTC: further Insights into Their Cooperative Action
Source: mBio. 2021 Oct 5;12(5):e02585-21. doi: 10.1128/mBio.02585-21 (PMC8546558; doi:10.1128/mBio.02585-21)
Supplement: TABLE S3 [file mbio.02585-21-st003.docx]

**Table S3 Oligonucleotides used in this study**

| **Primers** | **Sequence (5’**—**>3’)** |
| --- | --- |
| PQ1 | ATTATAAGGAGGCACTCAAAATGGATGAAGTGAAAGAATTCACATC |
| PQ2 | TTATTCATCATTATCCTCATATTGCTCTGAATAATAAAGTTC |
| PQ3 | GAGCAATATGAGGATAATGATGAATAAGGATCCGCTTTCTTTGAACCAAAATTAG |
| PQ4 | TTTGAGTGCCTCCTTATAATTTATTTTGTAGTT |
| PQ5 | CATTGTAGTATTCACGTAAGCAAATAACATGAAATGAGGACTAATAGATGGATGAAGTG |
| PQ6 | TTATTTGCTTACGTGAATACTACAATGACAAGTTG |
| PQ7 | TGATGAACATCATCACCATCACCATTAAGGATCCGCTTTCTTTGAACCAAAATTAG |
| PQ8 | TAATGGTGATGGTGATGATGTTCATCATTATCCTCATATTGCTCTGAATAATAAAG |
| PQ9 | CATTTCTGCTAGTTTGAATGCTGCCAGAAAAGC |
| PQ10 | AAACTAGCAGAAATGAAAATTGAAATATTATTTTCCGAAAGAGC |
| PQ11 | CAATATGAGGATAATGATGAAGGTAGCGGTGGAGGTGGCAG |
| PQ12 | TTACTTATAAAGCTCATCCATGCCGTG |
| PQ13 | CACGGCATGGATGAGCTTTATAAGTAAGGATCCGCTTTCTTTGAACCAAAATTAG |
| PQ14 | CTGCCACCTCCACCGCTACCTTCATCATTATCCTCATATTGCTCTGAATAATAAAGTTC |
| PQ15 | CTTTATAAGCATCATCACCATCACCATTAAGGATCCGCTTTCTTTGAACCAAAATTAG |
| PQ16 | ATGGTGATGGTGATGATGCTTATAAAGCTCATCCATGCCGTGAG |
| PQ17 | GGTGGAGGTGGCAGCCATCATCACCATCACCATTAACCAAATCAAAG |
| PQ18 | GCTGCCACCTCCACCGCTACCACGTCCTTCAATTTTGCTTACGTGAATACTACAATGAC |
| PQ19 | CATGACATTGACTATAAAGACGATGACGATAAATGAGGATCCGCTTTCTTTGAACC |
| PQ20 | ATCTTTATAGTCTCCGTCATGATCTTTATAGTCTTTCATGTATTCTTCCGAAACAAACAACC |
| PQ21 | TTGTCATGCTAGTATTCACGTAAGCAAAATTGAAGGAC |
| PQ22 | TGAATACTAGCATGACAAGTTGCTGTTTTCATGTTAC |
| PQ23 | AGCAACTGCACATGCTAGTATTCACGTAAGCAAAATTG |
| PQ24 | TAGCATGTGCAGTTGCTGTTTTCATGTTACAACCCATC |
| PQ25 | ATGGGTGCTAACATGAAAACAGCAACTGCACATG |
| PQ26 | CATGTTAGCACCCATCAGAGCTCCTGTTTTACAAC |
| PQ27 | CCCGGTGCAAAAACAGGAGCTCTGATGGGTG |
| PQ28 | TGTTTTTGCACCGGGTGTACATAGCGAAATAC |
| PQ29 | GCTACACCCGGTGCAAAAACAGGAGCTCTGATGGGTG |
| PQ30 | TGCACCGGGTGTAGCTAGCGAAATACTTGTAATGCGTGGTG |
| PQ31 | AAGGAGGCACTCAAAATGATAAAAAGTTCATTTAAAGCTCAACC |
| PQ32 | TTTGAGTGCCTCCTTATAATTTATTTTGTAGTTCC |
| PQ33 | TGAAGGACGTCATCATCACCATCACCATTAACCAAATC |
| PQ34 | GGTGATGATGACGTCCTTCAATTTTGCTTACGTGAATAC |
| PQ35 | TTTATTAGAACGAGAGCAGACTATAAAGATCATGACGGAGACTATAAAGATCATGACATT |
| PQ36 | TCTGCTCTCGTTCTAATAAAAGGCACTACAAC |
| PQ37 | CATCATCACCATCACCATTAAGCTTTCTTTGAACCAAAATTAGAAAACC |
| PQ38 | GTGATGGTGATGATGGCGTGGTGATGCACCTGAATCTTTC |
| PQ39 | ATTATAAGGAGGCACTCAAAATGGAAAAGGGGAGAGTTGCCGATGTTG |
| PQ40 | TTTGAGTGCCTCCTTATAATTTATTTTGTAGTTCC |
| PQ41 | GAAATGAATTATTCAGAGCAATATGAGGATAATGATG |
| PQ42 | TGAATAATTCATTTCATGTATTCTTCCGAAACAAACAACC |
| PQ43 | CATGACATTGACTATAAAGACGATGACGATAAATGAATTATTCAGAGCAATATGAGGATAATGATG |
| PQ44 | ATCTTTATAGTCTCCGTCATGATCTTTATAGTCTTTCATGTATTCTTCCGAAACAAACAACC |
| PQ45 | CATGACATTGACTATAAAGACGATGACGATAAATGACTGCAGGACCCAGCTTTCTTG |
| PQ46 | ATCTTTATAGTCTCCGTCATGATCTTTATAGTCTTTCATGTATTCTTCCGAAACAAACAACC |
| PQ47 | AAGGAGGCACTCAAAATGAGGATAATGATGAATAAAAAAAATATAAAAAGAAATG |
| PQ48 | TTTGAGTGCCTCCTTATAATTTATTTTGTAGTTCC |
| PQ49 | CATCACCATCACCATTGACTGCAGGACCCAGCTTTCTTG |
| PQ50 | ATGGTGATGGTGATGATGTTTCCTCTTCCCTCCTTTCAAAAAATCGTC |
| PQ51 | ATTATAAGGAGGCACTCAAAATGTTAGGTAATGAAGGGAGAGCATTTATAAGAG |
| PQ52 | TTTGAGTGCCTCCTTATAATTTATTTTGTAGTTCC |
| PQ53 | TGATGAACATCATCACCATCACCATTAAGGATCCGCTTTCTTTGAACCAAAATTAG |
| PQ54 | TAATGGTGATGGTGATGATGTTCATCATTATCCTCATATTGCTCTGAATAATAAAG |
| PQ55 | AAGAGAGGAAAAAACATGTTAGGTAATGAAGGGAGAGCATTTATAAGAGAG |
| PQ56 | GTTTTTTCCTCTCTTTTATGCTCTCGTTCTAATAAAAGGCACTAC |
| PQ57 | GAGAGCATAACATGAAATGAGGACTAATAGATGGATGAAGTG |
| PQ58 | TCATGTTATGCTCTCGTTCTAATAAAAGGCACTAC |
| PQ59 | AAGAGAGGAAAAAACATGTTAGGTAATGAAGGGAGAGCATTTATAAGAGAG |
| PQ60 | CATGTTTTTTCCTCTCTTTATTTTTATAAGCTATTTAGC |
